# Age-Related Differences in the Late Positive Potential during Emotion Regulation between Adolescents and Adults

**DOI:** 10.1038/s41598-019-42139-4

**Published:** 2019-04-05

**Authors:** Xinmei Deng, Biao Sang, Yixuan Ku, Liyang Sai

**Affiliations:** 1 College of Psychology and Sociology, Shenzhen University, Shenzhen, China; 2 Shenzhen Key Laboratory of Affective and Social Cognitive Science, Shenzhen, China; 3 Shanghai Academy of Educational Science, Shanghai, China; 4 School of Psychology and Cognitive Science, East China Normal University, Shanghai, China; 5 Hangzhou Normal University, Hangzhou, China

## Abstract

The late positive potential (LPP) has been well documented in predicting the effect of emotion regulation in previous developmental literature. However, few studies have examined age-related changes in emotion regulation from adolescence to adulthood using this biomarker. To test this, Reactivity and Regulation-Image Task was used to test 18 young adolescents and 22 adults to examine the modulation of LPP during emotion regulation. Results revealed that (a) on the behavioral level, adults reported higher intensity of emotional experience than adolescents when they were asked to use up-regulation. Down-regulation showed no age effect for self-reported rating; (b) adolescents showed higher amplitudes of LPP than adults when using different regulatory strategies in all windows; (c) In late time window, regulation effect was larger when using up-regulation strategy than down-regulation strategy for adolescents, while the difference between the two strategies was negligible for adults. (d) In early time window, reactivity effect was larger in negative conditions than in positive conditions for adolescents, while the difference between the two conditions was again negligible for adults. Differences in the amplitudes and time courses of LPP during emotion regulation between adolescents and adults suggested that age-related changes in emotion regulation may occur during adolescence.

## Introduction

Emotion regulation is a process that integrates physiological, cognitive, and behavioral components^1^. It refers to how effectively individual regulates emotional responses. Emotional reactivity refers to how strong one’s emotional response is to affective versus neutral stimuli^2^. It is supported by neural systems involved in both affective information and executive control processes^3^. Emotion regulation develops remarkably through adolescence^4^. It reflects the tendency of emotional responses to stimuli combined with the ability to modify those responses^2,5^. Investigating age differences in the physiological components of emotion regulation (e.g., scalp-recorded event related potentials, ERPs) is important in terms of understanding the emotional development during adolescence.

## Reactivity Effect and Regulation Effect of Emotion Regulation

According to the extended process model of emotion regulation, emotional reactivity effect and emotion regulation effect are two major indexes that indicate the process of emotion regulation^1^. Emotional reactivity is the threshold and intensity of individuals’ response to emotional stimuli^2,3^. Emotion regulation effect is defined as the effectiveness of the regulation of ones’ emotional response^2,5^. That is to say, when individuals want to enhance their feelings of joy or sadness, they can increase the intensity of their emotional experience. Conversely, when individuals want to reduce their joy or sadness, they can reduce the emotional experience to how calm it is.

Electroencephalogram (EEG) represents brain’s spontaneous brain activity during processing of the stimulus. When the EEG is time-locked to specific events and presentation of the stimulus, the resulting voltage modulations over time are referred to as event related potentials^6^. A series of scalp-recorded event related potential (ERP) studies have demonstrated the modulation of specific ERP components during emotion regulation^5,7^. For example, the late positive potential (LPP) is a superior-posterior positivity starting from about 300–400 ms after stimuli onset and is considered to be an important biomarker for emotion regulation^7^. Prior studies suggest that the amplitude of LPP would change based on different regulatory strategies chosen by participants in different emotional conditions^6,8^. The dual role of reactivity and regulation effect during emotion regulation could be indexed by the modulation of LPP^5^. For example, previous studies indicated that LPP during response to emotionally arousing stimuli increased than that to neutral stimuli^9,10^. The increase is even larger when emotional stimuli evoke high arousal levels, such as with the presence of erotic videos and disgusting pictures^9^. The reactivity effect of emotion regulation reflects an emotionally aroused process during emotion regulation^11^. These studies suggest that LPP reflects the processing of emotionally arousing features of the stimulus.

Apart from the reactivity effect of LPP, recently a number of studies have suggested that emotion regulation effect could also be indexed by the modulation of LPP amplitude^12^. For example, LPP decreased when participants down-regulated their emotions, compared with participants who passively viewed the emotional stimuli (e.g. participants were instructed to view the pleasant image from a detached, uninvolved perspective)^13,14^. Comparing with the passive view condition, LPP increased when participants up-regulated their emotion (e.g., participants were instructed to view the picture from an attached or first-person perspective as someone personally invested)^15^. The sensitivity of LPP amplitude to emotion regulation instruction may suggest that individuals could recruit their cognitive resources to modify their emotional experience^5,11^. Therefore, the regulation effect of emotion regulation reflects the modification of emotion experience under emotionally demanding conditions^7^. Together, the dual role of reactivity and regulation effect during emotion regulation could be both indexed by the modulation of LPP^5^.

## Developmental Differences in the LPP

Although efforts have been made to characterize developmental differences in LPP during emotion regulation from early childhood to late adulthood^5,7^, little has been examined about such age-related changes during adolescence.

In terms of the reactivity effect of emotion regulation, for example, previous work has shown that toddlers exhibit increases in LPP in response to negative emotional stimuli compared with neutral stimuli^7^. These increases in LPP have been found in even 7 months old children^16^. However, a study of adults’ reactivity effect of negative emotional stimuli suggested that the increases in LPP magnitude declined linearly with age across most of the adulthood^17^. Based on the previously mentioned work with the sample of children and adults, it would be natural to consider the age impact on reactivity effect to different emotional stimuli during adolescents’ time, which is an important interconnecting period in between childhood and adulthood.

As to the development of regulation effect, LPP has been well documented as a typical biomarker in the sample of children and adults in previous literature. As stated before, emotion regulation modulates the amplitude of LPP^14^. The significant decrease in LPP was observed when participants (*M*_*age*_ = 20.7) were instructed to regulate their emotions^11^. However, in the younger population, prior research has found that usage of reappraisal to down-regulate the significance of unpleasant emotional materials did not modulate LPP in 5 to 7-year-olds^18^. Different findings between adults and children suggest that the ability to effectively regulate emotions might be developed and acquired starting from childhood to early adulthood. Neuroimaging literature has further documented that emotion regulation ability continues to develop during middle and late childhood^19^. Thus, it is important to examine LPP in adolescents to identify the sensitive period of emotion regulation development between childhood and early adulthood.

More importantly, adolescence is often depicted as a period of ‘storm and stress’, characterized by mood fluctuations^4^. To cope with various social challenges and psychological disturbances, adolescents may have a greater demand for emotion regulation than younger children and adults^20^. Therefore, understanding how emotion regulation develops during this important period can facilitate the understanding of socio-emotional development.

## The Present Study

The present study thus investigated the age-related differences between adolescents and adults in the LPP for both emotional reactivity and regulation in the context of up-regulating and down-regulating the emotional pictures. Most previous studies on emotion regulation focused on emotion down-regulation with little or no concentration on emotion up-regulation, which is also important for individuals in some occasions. For instance, individuals would seek to increase their emotions of anger during a competitive task for a utilitarian purpose^21,22^. Thus, the present study investigated age-related differences between adolescents and adults for both down-regulation as well as up-regulation.

LPP is an ERP that reflects facilitated attention to emotional stimuli^7^ and emotion regulation^23^. The early LPP reflects the relatively early initial reactivity to an emotional stimulus and the later LPP reflects the more regulated stages of affective processing^11^. Previous studies demonstrated the age-related differences in the time course of emotion regulation between children and adults. For example, children showed increased amplitude in LPP (reactivity effect) to emotional image compared to neutral by 500 ms. On the contrary, the reactivity effect of adults was presented as early as 200–300 ms^7^. Also, the regulation effect of young children was presented later than adults^5^. Thus, it would be more meaningful to divide LPP into multiple time windows that reflect different stages of emotion regulation. Also, LPP shows a spatial shift over time. LPP is normally maximal at posterior-superior recording sites in the early window and shifts to being comparable at posterior and anterior recording sites during later time windows^5^. Previous studies suggested that both adults and young children showed such spatial shift over time^24^. However, to our knowledge, spatial shift over time has not been examined yet in the adolescent population. Therefore, exploring age-related differences in the time course (different time windows of LPP) and topography of the LPP might provide insights into the development of emotion regulation in adolescence.

Up-regulation is a type of regulatory strategy that increases and maximizes the degree of emotion experience and amplifies behavioral and facial responses. Conversely, down-regulation was defined as a regulatory strategy that decreases and minimizes the intensity of emotional experience, and weakens behavioral and facial responses^3,14^. The hypotheses of the present study are: (1) Because of the structural changes and the functional changes in the developing brain, adolescents are more emotionally reactive than children and adults^25^. This notion was presented by Stephanou *et al*.’s study of adolescents and young adults (age 15–25)^26^. When using reappraisal to down-regulate aversive social imagery, younger participants exhibited greater activation of temporal- occipital scalp area than their older counterparts. In another ERP study, a decrease in LPP magnitude was found in response to emotional stimuli compared to the neutral stimuli for adulthood period^17^. Thus, we expected that adolescents (compared to adults) would report a higher LPP when they passively view different emotional stimuli (higher reactivity effect). (2) Behavioral and neuroimaging measures indicate that individuals become more effective in modulating their emotions starting from late childhood through adolescence to early adulthood^2,27,28^. We expected that adults (compared to adolescents) were expected to have a higher regulation effect. Specifically, since adults require less neural resources for emotional information processing and show less activation in the cortical regions than adolescents^8^, we expected that LPP during emotion regulation would be larger in adolescents than that in adults. (3) The early LPP reflects the initial reactivity to an emotional stimulus and the later LPP reflects the recruitment of prefrontal cortical resources associated with effective cognitive control^5,11^. Therefore, we expected the age-related differences in the reactivity effect would be presented in the relatively early time window and the differences in the regulation effect would be presented in the later time window. (4) Previous studies suggested that both adults and young children showed a spatial shift over time from predominantly posterior cortical activity to equally distributed anterior activity^24^. Thus, we also expected the same spatial shift in adolescents.

## Material and Methods

### Participants

Participants were 18 Chinese adolescents and 22 Chinese adults. Adolescence is a transitional stage of physical and psychological development that generally occurs during the period from puberty to adulthood. Adolescence usually referred to teenage years^29^. Participants were recruited via fliers that invited healthy volunteers to participate in studies of emotion that were distributed in two junior middle schools and one university. Before participating in the study, adolescent participants, parents of the adolescent participants, and adult participants were thoroughly introduced to the experiment and written informed consents were taken. All subjects were financially compensated for participation (100 China Yuan). The research protocol was approved by the Institutional Reviewing Board, East China Normal University. Experiments were performed in accordance with scientific research guidelines and regulations. Data from 5 participants were excluded due to noisy EEG recordings. The final sample consisted of 17 adolescents (10 male, 7 female; *M* = 13.29, *SD* = 0.47) and 18 adults (10 male, 8 female; *M* = 23.78, *SD* = 2.41). All of the participants came from urban communities. In the sample, 47.1% of the adolescents and 38.9% of the adults were the only child of their parents; others had one or more siblings. Approximately 64.7% of fathers and 58.8% of mothers in the adolescent group, and 55.6% of fathers and 55.5% of the mothers in the adults group had received a college education. Other parents had received an education of high school or lower. All participants were in good neurological and psychiatric condition. No participant had a history of neurological or psychiatric disorder, determined by self and/or parent report.

### Materials

The stimuli were 40 positive, 40 negative, and 40 neutral pictures from the Chinese Affective Picture System^30^. The pictures are appropriate for adolescents. Analysis of variance (ANOVA) showed that these three types of pictures significantly differed in terms of valence ratings (*F*(2, 119) = 1384.39, *p* < 0.001, *M* = 7.44, *SD* = 0.20 for positive pictures; *M* = 5.39, *SD* = 0.70 for neutral pictures; *M* = 2.35, *SD* = 0.21 for negative pictures; for all the paired comparisons *p* < 0.001). Positive and negative pictures were further matched in terms of arousal ratings for negative pictures (*M* = 5.59, *SD* = 0.29) and for positive pictures (*M* = 5.50, *SD* = 0.25) were more arousing than neutral pictures (*M* = 3.63, *SD* = 0.84), *ps* < 0.001. The negative picture set included unpleasant social situations and fearful animals. The positive picture set included pictures of lovely animals, appetizing food and pleasant social situations. The neutral pictures had depictions such as household objects. The pictures (330 × 340 pixels) were presented in color by a 17-in monitor. Participants viewed the pictures from a distance of approximately 65 cm and about 35◦ of visual angle—horizontally and vertically.

### Procedure

**Figure 1:**
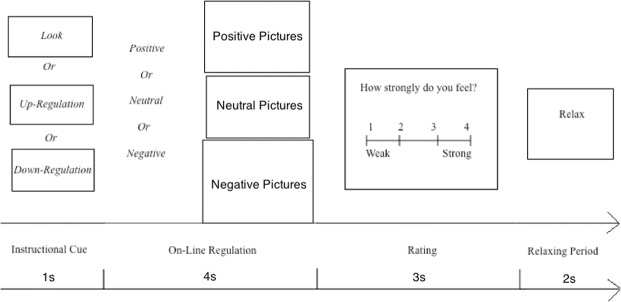
Procedure of Each Trial.

The present study utilized the Reactivity and Regulation-Image Task (REAR-I Task). The task was presented using E-Prime software (Psychological Software Tools, Pittsburgh, PA). This task has been shown to successfully examine both emotional reactivity and emotion regulation in a broad age range in a series of prior studies^2,3,31–33^. In the orientation section before the formal experiment, participants were instructed on task procedures. Instructions for the present study were adapted from Ochsner and Moser’s studies^3,15^. They were told that in the coming experiment, they would watch several pictures that may arouse different emotions, and they would need to regulate their emotions according to the three possible instructions—“↑Up-Regulation↑” or “↓Down-Regulation↓”or “Look”, which meant up-regulating their emotions, down-regulating their emotions, or naturally reacting to the pictures, respectively. Then, the definition of up-regulation and down-regulation were explained. Following this, the examiner gave each participant a list of samples of the two types of regulation, and thoroughly explained the usage of up and down-regulation. For Look trials, participants were asked to view the pictures and respond naturally. For Up-Regulation trials, participants were instructed to view the picture from the first-person perspective as someone personally participating in the pictured events. For the Down-Regulation trials, participants were instructed to view the picture either from a third-person perspective as someone with no personal association to the pictured event/object or as if it were fake. As the primary manipulation check, the experimenter asked participants how they responded on the task in different experimental conditions to determine whether or not participants understood and followed the instructions. Participants’ responses indicated that all of them understood the instructions and regulated their emotions according to the instructions. Electrode cap was attached and the experiment began (see Fig. 1).

An experimental trial began with the instruction of emotional regulatory strategies (“↑Up-Regulation↑”/“↓Down-Regulation↓”/“Look”) for one second. One positive, negative, or neutral picture was then shown for four seconds. Participants were told to view the picture and to regulate their emotional reactivity to the picture by using the instructed strategy. The trial was thereafter ended by asking participants to rate their current intensity of emotion on a 4-point scale (1 = have very weak emotion, 4 = have very strong emotion) via a button press. The inter-trial-interval was 2 seconds.

The current task consisted of four experimental blocks (360 experimental trials in total). In each block, the participants were instructed to up-regulate, down-regulate and view neutrally both positive and negative pictures. There were 9 experimental conditions. Each block consisted of 10 neutral pictures, 10 positive and 10 negative pictures. Each picture was repeated three times for the three regulatory instructions respectively. With this, there were 90 trials in each block. The order of trials was random within each block.

### EEG Recording and Signal Processing

EEG was recorded with a 28-channel amplifier (BrainAmp, Brain Products, Germany) and sampled at 512 Hz. Electrode gel was applied to produce an impedance of less than 10 kΩ. FCz was used as on-line recording reference. Data were then re-referenced offline to the averaged mastoid references and bandpass filtered from 0.1 Hz to 30 Hz. Eye movement and blink artifacts were corrected by using the independent component analysis (ICA) algorithm implemented in Brain Vision Analyzer 2.0 (Brain Products, Germany). Data were segmented in epochs from 250 ms before the onset of stimuli until 1500 ms after the onset. The mean amplitude from the 250 ms interval prior to the stimulus was used for baseline correction. Trials with artifacts exceeding ±50 μV were excluded from further analysis.

Analysis of LPP was divided into two sections. First, we examined the age differences in the amplitudes of LPP for each time window. To examine the age differences in the amplitude of LPP, ERPs were averaged according to the stimuli type (Valence: positive, negative and neutral) and regulation instruction (Strategy: up-regulation, down-regulation and no-regulation), yielding nine conditions.

Secondly, we examined the age differences in the emotional reactivity effects and regulation effects. We subtracted LPP magnitude for neutral Look condition from LPP magnitude for both positive and negative Look conditions to be used as the index of emotional reactivity effects. For the regulation effects, we subtracted LPP magnitude for Look condition from LPP magnitude for down and up-regulation (as shown below) to be used as the index of regulation effects.

***Reactivity Effects:***

*Positive Reactivity Effects* = *LPP (Look Positive)*–*LPP (Look Neutral*)

*Negative Reactivity Effects* = *LPP (Look Negative)*–*LPP (Look Neutral*)

***Regulation Effects:***

*Positive Up-regulation Effects* = *LPP (Positive Up-regulation)*–*LPP (Look Positive)*

*Positive Down-regulation Effects* = *LPP (Positive Down-regulation)*–*LPP (Look Positive)*

*Negative Up-regulation Effects* = *LPP (Negative Up-regulation)*–*LPP (Look Negative)*

*Negative Down-regulation Effects* = *LPP (Negative Down-regulation)*–*LPP (Look Negative)*

Based on the previous research^7,11^ and visual inspection, LPP was evaluated as the average activity in 6 regions of interest (ROIs) covering the left-anterior (Fp1, F3, F7, FC5), medial- anterior (Fz, FC1, FC2), and right- anterior (Fp2, F4, F8, FC6), left-posterior (CP5, P3, P7, O1), medial-posterior (CP1, CP2, Pz, Oz), and right-posterior (CP6, P4, P8, O2) recording sites. The ERP waveforms were quantified by mean amplitude measures in three time windows following stimulus onset: 400–700 ms (early window), 700–1000 ms (middle window), and 1000–1500 ms (late window). These were classified as the early LPP, middle LPP and late LPP respectively^11^. Originally there were 40 epochs per condition per subject. There were 360 experimental trials in the study in total. The mean number of valid epochs averaged per condition per subject ranged from 27 (67.5%) to 34 (85%). In most ERP studies with children population, the mean numbers of valid epoch range from 15 to 30^5,7^. The mean numbers of valid epochs averaged per condition in our study were higher than normal standards of the ERP studies with children population. Standard errors of the ERPs were calculated to represent the measures of inter-subject variability. In the early time window, standard errors of ERPs ranged from 0.243 to 0.989 per participant for adolescents and standard errors of ERPs ranged from 0.148 to 0.542 per participant for adults. In the middle time window, standard errors of ERPs ranged from 0.190 to 0.771 per participant for adolescents and standard errors of ERPs ranged from 0.133 to 0.496 per participant for adults. In the late time window, standard errors of ERPs ranged from 0.176 to 1.118 per participant for adolescents and standard errors of ERPs ranged from 0.122 to 0.421 per participant for adults.

### Statistical Analyses

Self-ratings of emotion experience intensity in different experimental conditions were examined by using a 2 (Age: adolescents vs. adults) × 3 (Valence: neutral vs. positive vs. negative) × 3(Strategy: no-regulation vs. up-regulation vs. down-regulation) repeated measures ANOVA.

Subject averages and grand averages were calculated for each ROI and experimental conditions. When conducting statistical analysis, we included one ERP value per subject (average) per electrode. For the LPP in each time window, a 2 (Age: Adolescents vs. Adults, between factor) × 3 (Valence: within factor) × 3 (Strategy: within factor) × 3 (Laterality: left vs. medial vs. right) × 2 (Anterior vs. Posterior) repeated measures ANOVA was conducted separately.

For the regulation effect on LPP in each time window, a 2 (Age: adolescents vs. adults) × 2 (Valence: positive vs. negative) × 2 (Strategy: up-regulation vs. down-regulation) × 3 (Laterality: left vs. medial vs. right) × 2 (Anterior vs. Posterior) repeated measures ANOVA was conducted separately. For the reactivity effect of LPP in each time window, a 2 (Age: adolescents vs. adults) × 2 (Valence: positive vs. negative) × 3 (Laterality: left vs. medial vs. right) × 2 (Anterior vs. Posterior) repeated measures ANOVA was conducted separately.

Greenhouse-Geisser corrections were applied to *p* values when the assumption of sphericity for ANOVA was violated. Greenhouse-Geisser epsilons were reported where appropriate. The significance level was set at *p* < 0.05. In the analysis, *p* values were adjusted with the Bonferroni correction for multiple post hoc comparisons.

## Results

### Behavioral Results

**Figure 2:**
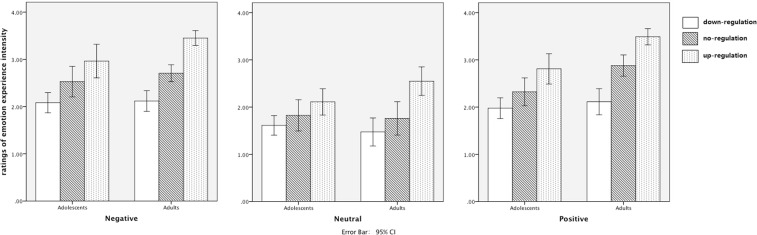
Behavioral results for different experiment condition between adolescents and adults.

There were significant main effects of Valence (*F*(2, 32) = 70.13, *p* < 0.001, ${\eta }_{p}^{2}$ = 0.68), and Strategy (*F*(2, 32) = 116.62, ε = 0.69, *p* < 0.001, ${\eta }_{p}^{2}$ = 0.78). Ratings of emotion experience intensity differed as a function of regulation strategy (up-regulation > no-regulation > down-regulation, all *p* < 0.001). No significant main effect of Age was found (*F*(1, 33) = 3.75, ε = 0.74, *p* = 0.06, ${\eta }_{p}^{2}$ = 0.10). There was also a significant Age × Strategy (*F*(2, 32) = 8.08, *p* < 0.01, ${\eta }_{p}^{2}$ = 0.20). Post-hoc tests indicated that under the up-regulation conditions, adults’ subjective ratings of emotion experience intensity were higher than the ratings by adolescents (*p* < 0.05, as shown in Fig. 2). However, there was no significant age-related difference in down-regulation (*p* > 0.05). No significant Age × Strategy × Valence interaction was found (*F*(4, 30) = 2.54, *p* = 0.056, ${\eta }_{p}^{2}$ = 0.07).

Furthermore, relative changes (subtraction between up and down-regulation with no-regulation) were compared between adolescents and adults. Results showed that the relative change in the negative up-regulation was larger for adults than adolescents (*M*_*adults*_ = 0.74, *M*_*adolescents*_ = 0.43, *t*(33) = −2.41, *p* = 0.022). Also, the relative change in the positive down-regulation was larger for adults than adolescents (*M*_*adults*_ = 0.77, *M*_*adolescents*_ = 0.35, *t*(33) = −2.86, *p* = 0.007). There were no significant differences in the negative down-regulation condition (*M*_*adults*_ = 0.59, *M*_*adolescents*_ = 0.45, *t*(33) = −1.00, *p* = 0.323) and positive up-regulation (*M*_*adults*_ = 0.61, *M*_*adolescents*_ = 0.49, *t*(33) = −0.97, *p* = 0.340). The age-related differences in the relative changes between regulation and no-regulation suggested that adults were more capable of negative up-regulation and positive down-regulation than adolescents.

### ERP results

#### Differences in the amplitude of LPP

For LPP in the early time window, the main effect of Strategy was not significant, *F*(2, 64) = 1.47, *p* = 0.24, ${\eta }_{p}^{2}$ = 0.04. The main effect of Valence was significant, *F*(2, 64) = 82.53, *p* < 0.001, ${\eta }_{p}^{2}$ = 0.72. LPP was larger for both positive and negative emotional stimuli than neutral stimuli (both *ps* < 0.001). LPP was larger for negative emotional stimuli than positive emotional stimuli (*p* < 0.001). The main effect of Laterality was significant, *F*(2, 64) = 17.79, *p* < 0.001, ${\eta }_{p}^{2}$ = 0.36. LPP was larger for right and left recording sites than medial recording sites (both *ps* < 0.001). The main effect of Anterior-Posterior was not significant (*F*(1, 33) = 4.06, *p* = 0.052, ${\eta }_{p}^{2}$ = 0.11). The main effect of Age was significant, *F*(1, 33) = 30.38, *p* < 0.001, ${\eta }_{p}^{2}$ = 0.49. LPP was larger for adolescents than adults (*p* < 0.001). There was a significant interaction in Laterality × Age, *F*(2, 64) = 3.94, *p* = 0.026, ${\eta }_{p}^{2}$ = 0.11. For both group, LPP was larger for right and left recoding sites than medial recording sites (adolescents: *p* < 0.001; adults: *p* = 0.014). There was a significant interaction in Valence × Age, *F*(2, 64) = 7.06, *p* = 0.004, ${\eta }_{p}^{2}$ = 0.18. For both positive and negative emotional condition, LPP was larger for adolescents than adults (both *ps* < 0.001). However, there was no significant age-related difference in neutral condition (*p* > 0.05). There was a significant interaction in Valence × Laterality × Age, *F*(4, 128) = 4.25, *p* = 0.005, ${\eta }_{p}^{2}$ = 0.117. In the positive, negative and neutral emotional condition, LPP was larger for right and left recording sites than medial recording sites in adolescents group (both *ps* < 0.001). In the positive emotional condition, adults’ LPP was larger for left recording sites than medial recording sites in adults group (*p* = 0.008). In the negative emotional condition, adults’ LPP was larger for left recording sites than medial and right recording sites in adults group (*p*_*medial*_ < 0.001, *p*_*right*_ = 0.049). There was no significant difference between different recording sites in neutral condition in adults group (all *ps* > 0.05).

For LPP in the middle time window, the main effect of Strategy was not significant, *F*(2, 64) = 1.32, *p* = 0.70, ${\eta }_{p}^{2}$ = 0.01. The main effect of Valence was significant (*F*(2, 64) = 27.17, *p* < 0.001, ${\eta }_{p}^{2}$ = 0.46). LPP was larger for negative emotional stimuli than neutral stimuli (*p* < 0.001). LPP was larger for positive emotional stimuli than neutral stimuli (*p* < 0.001). LPP was larger for negative emotional stimuli than positive emotional stimuli (*p* = 0.001). The main effect of Laterality was not significant, *F*(2, 64) = 1.64, *p* = 0.21, ${\eta }_{p}^{2}$ = 0.05. The main effect of Anterior-Posterior was significant, *F*(1, 33) = 4.56, *p* = 0.041, ${\eta }_{p}^{2}$ = 0.13. LPP was larger for posterior recording sites than anterior recording sites. The main effect of Age was significant (*F*(1, 33) = 17.40, *p* < 0.001, ${\eta }_{p}^{2}$ = 0.35). LPP was larger for adolescents than adults (*p* < 0.001). There was a significant interaction in Valence × Age (*F*(2, 64) = 4.57, *p* = 0.022, ${\eta }_{p}^{2}$ = 0.13). For both group, LPP was larger for negative emotional stimuli than positive and neutral stimuli (adolescents: both *ps* < 0.001; adults: *p*_*positive*_ = 0.048, *p*_*neutral*_ = 0.044). For adolescents, LPP was larger for positive emotional stimuli than neutral stimuli (*p* = 0.001). There was no significant difference between positive emotional stimuli than neutral stimuli in adults. There was a significant interaction in Valence × Anterior-Posterior × Strategy (*F*(4, 132) = 2.83, *p* = 0.03, ${\eta }_{p}^{2}$ = 0.08). In the positive condition, LPP was larger for posterior recording sites than anterior recording sites when using different types of regulatory strategies (*p*_*up-regulation*_ < 0.001, *p*_*down-regulation*_ = 0.005, and *p*_*down-regulation*_ = 0.021). There was no significant difference between posterior and anterior recording sites in negative condition when using different types of regulatory strategies (all *ps* > 0.05).

**Figure 3:**
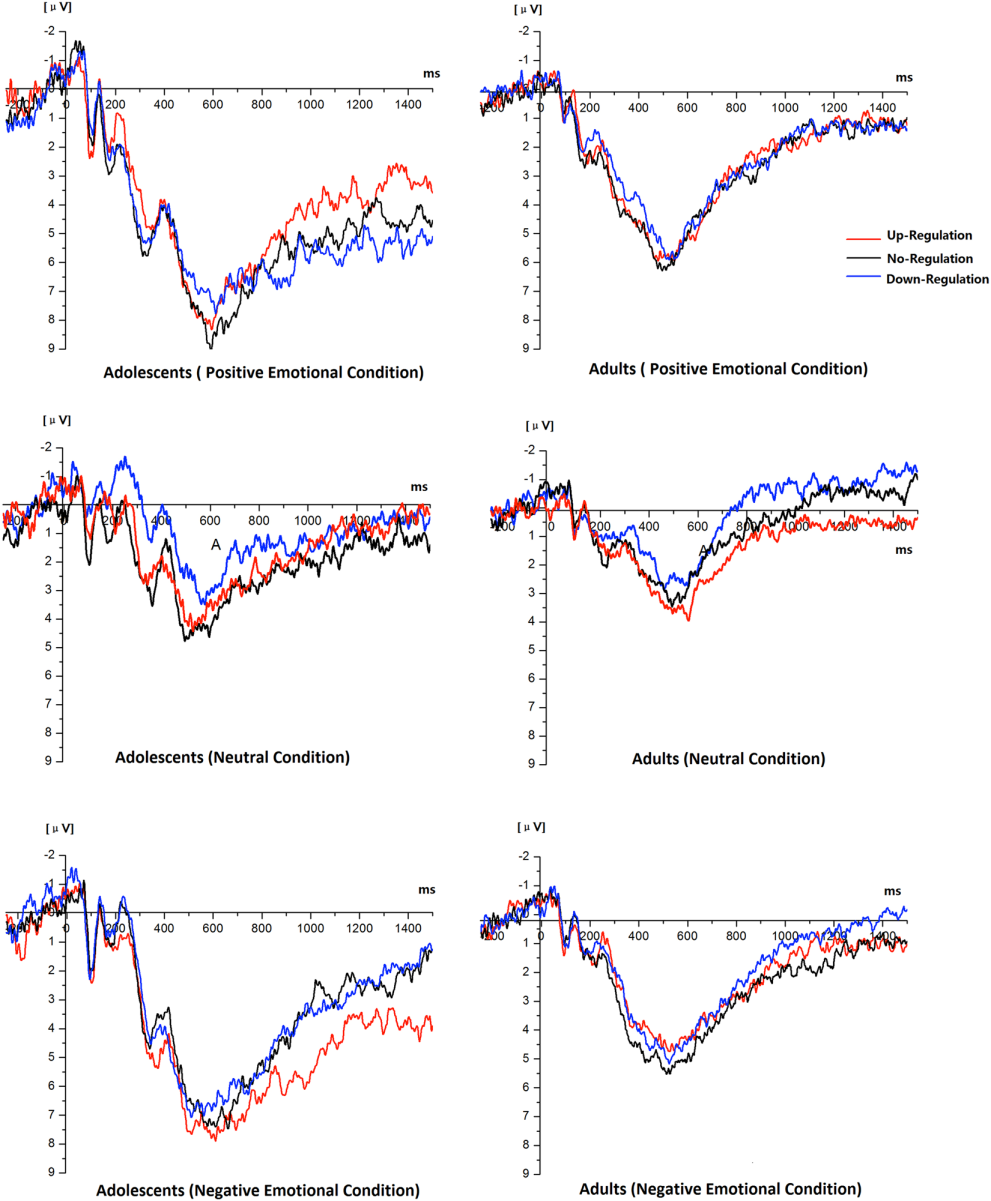
The ERP wave forms at the medial-posterior recoding sites for different experimental conditions between adolescents and adults.

For LPP in the late time window, the main effect of Valence was significant for LPP (*F*(2, 64) = 4.96, *p* = 0.01, ${\eta }_{p}^{2}$ = 0.13). Both positive and negative emotional stimuli induce larger LPP than neutral stimuli (*p* = 0.016), but there was no significant difference between positive and negative stimuli. The main effect of Age was significant (*F*(1, 33) = 15.025, *p* < 0.001, ${\eta }_{p}^{2}$ = 0.32). LPP was larger for adolescents than that for adults. No other main effect or interaction reached significant level. ERP wave forms at the medial-posterior recording sites for positive, neutral and negative emotion regulation conditions between adolescents and adults are shown in Fig. 3. Other main effects, two-, three- and four-way interactions involving Age did not reach significance (details about the test statistics are shown in the supplementary materials).

#### Differences in the Regulation Effect

For the decreases in LPP when using different regulatory strategy (the regulation effect) in the early time window, no significant main effect was found (all *F* ≤ 0.96, *p* ≥ 0.12, ${\eta }_{p}^{2}$ ≤ 0.007). There was a significant interaction in Age × Anterior-Posterior, *F*(1, 33) = 8.07, *p* = 0.008, ${\eta }_{p}^{2}$ = 0.20. For adults, the regulation effect was larger for anterior recording sites than posterior recording sites (*p* = 0.009). However, for adolescents, there was no significant difference between anterior and posterior recording sites (*p* > 0.05). For regulation in the middle time window, no significant main effect or interaction was found (all *Fs* ≤ 2.93, *ps* ≥ 0.097, ${\eta }_{p}^{2}$ ≤ 0.008). For regulation effect in the late time window, no significant main effect was found (all *F* ≤ 1.40, *p* ≥ 0.20, ${\eta }_{p}^{2}$ ≤ 0.04). There was a significant interaction in Age × Strategy, *F*(1, 33) = 7.90, *p* = 0.008, ${\eta }_{p}^{2}$ = 0.20. For adolescents, the regulation effect was larger for up-regulation strategy than for down-regulation strategy (*p* = 0.01). However, for adults, there was no significant difference between up- and down-regulation (*p* > 0.05).

Other main effects, two-, three- and four-way interactions involving Age did not reach significance (details about the test statistics are shown in the supplementary materials).

#### Differences in the Reactivity effect

For the increases in LPP when viewing different emotional valenced stimuli (the reactivity effect) in the early time window, the main effect of Valence was significant, *F*(1, 33) = 20.74, *p* < 0.001, ${\eta }_{p}^{2}$ = 0.39. The reactivity effect was larger in the negative emotional condition than in the positive emotional condition (*p* < 0.001). The main effect of Laterality was significant, *F*(2, 64) = 4.65, *p* = 0.02, ${\eta }_{p}^{2}$ = 0.13. The reactivity effect was larger in the left recording sites than in the medial recording site (*p* = 0.03). There was a significant interaction in Age × Laterality, *F*(2, 64) = 4.21, *p* = 0.02, ${\eta }_{p}^{2}$ = 0.12. For adolescents, the reactivity effect was larger in the right recording sites than the left (*p* = 0.008) and medial recording sites (*p* = 0.041). For adults, the reactivity effect was larger in the left recording sites than the medial recording sites (*p* = 0.047). There was a significant interaction in Age × Valence, *F*(1, 33) = 4.25, *p* = 0.04, ${\eta }_{p}^{2}$ = 0.12. For adolescents, the reactivity effect was larger in the negative emotional conditions than the positive emotional conditions (*p* < 0.001). However, for adults, there was no significant difference between negative and positive emotional conditions (*p* = 0.08).

For the reactivity effect in the middle time window, the main effect of Laterality was significant, *F*(2, 64) = 0.09, *p* = 0.91, ${\eta }_{p}^{2}$ = 0.003. There was a significant interaction in Age × Valence × Anterior-Posterior, *F*(1, 33) = 6.56, *p* = 0.02, ${\eta }_{p}^{2}$ = 0.17. For adolescents, the reactivity effect was larger in the negative emotional conditions than in the positive emotional conditions in the anterior recording sites (*p* = 0.038). However, for adults, there was no significant difference between negative and positive emotional conditions (*p* > 0.05). For adolescents, the reactivity effect was larger in the anterior recording sites than in the posterior recording sites in the negative emotional conditions (*p* = 0.015). However, for adults, there was no significant difference between anterior and posterior recording sites in the negative emotional conditions (*p* > 0.05).

For the reactivity effect in the late time window, no significant main effect or interaction was found (all *F* ≤ 1.29, *p* ≥ 0.28, ${\eta }_{p}^{2}$ ≤ 0.04).

**Figure 4:**
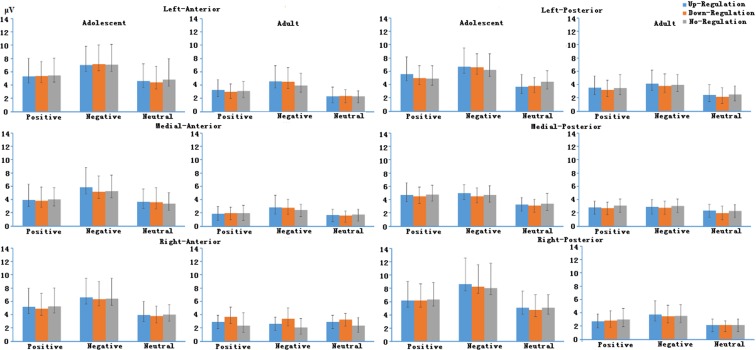
LPP averages in early time windows in different experimental conditions between adolescents and adults.

**Figure 5:**
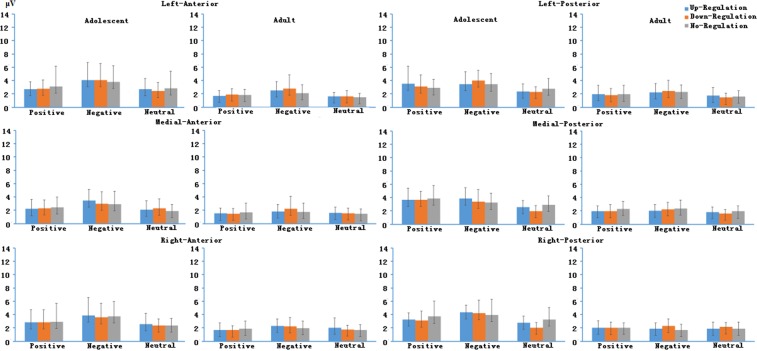
LPP averages in middle time windows in different experimental conditions between adolescents and adults.

**Figure 6:**
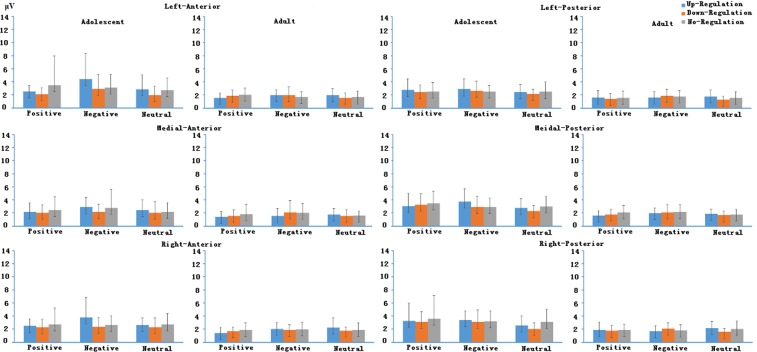
LPP averages in late time windows in different experimental conditions between adolescents and adults.

Other main effects, two-, three- and four-way interactions involving Age did not reach significance (details about the test statistics are shown in the supplementary materials). Means of LPP magnitudes in different time windows in positive, neutral and negative emotion regulation conditions between adolescents and adults are shown in Figs 4, 5 and 6.

## Discussion

A growing body of research indicates that LPP is a useful neural index for assessing age-related changes in emotion regulation^5,7,11^. The present study was the first to investigate the age-related differences between adolescents and adults in terms of the LPP modulations during emotion regulation processing. Results showed that the main effect of Valence was significant in all time windows. Generally, LPP was larger for emotional stimuli than neutral stimulus. In addition, consistent with the results from the prior study in the elderly population^17^, we found that amplitude of LPP decreased with development: larger amplitudes of LPP were found in adolescents than in adults.

LPP is a typical EEG component in the studies of emotion regulation processing. The modulation of LPP reflects the involvement of different neural systems in the time course of emotion regulation processing^13,34^. Recently, it has been demonstrated that LPP reflects the facilitated attention to emotion and the usage of cognitive control^35^. Also, reactivity and regulation effect during emotion regulation could be indexed by the modulation of LPP^5^. Evidence from the developmental cognitive neuroscience shows that there are steady changes in the structural and functional development of the human brain during adolescence^25^. The ability to regulate emotions continues to develop between adolescence and adulthood^35^. Adolescents are more emotionally reactive than children and adults^4^. In the present study, for adolescents, the reactivity effect was larger in the negative emotional conditions than in the positive emotional conditions. However, for adults, there was no significant difference between negative and positive emotional conditions. It is in line with the previous findings that adolescents are more vulnerable to negative information^2^. It could be concluded that adolescence might be a traditional period for researchers to explore the potential developmental changes in emotion regulation beginning from childhood to adulthood. Therefore, examining the differences in the ERPs during emotion regulation process between adolescents and adults is necessary.

As expected, the present study found that the LPP during emotion regulation processing was larger in adolescents than in adults. This result is in line with the prior studies which suggest that adults require less neural resources for cognitive control and emotional information processing and show less activation in the anterior recording sites than their younger counterparts during emotional processing (e.g., left lateralized ventro-lateral PFC, insula and right inferior parietal lobe, bilateral ventral prefrontal cortex, the right parietal lobe, and right dorsolateral prefrontal cortex)^36,37^. Due to development of brain socially, such neural efficiency improves with age. People acquire the ability to utilize fewer neural and cognitive resources when regulating emotions^8,35^.

In addition, the amplitude of LPP is not only an indicator of the involvement of neural resources: previous studies have shown that the modulation of LPP also marked emotion regulation processing. For example, the amplitude of LPP will change (reduce or enhance) following different emotion regulation strategies^11,13–15^. These findings suggest that LPP indicates processes that individuals use cognitive control to regulate emotion^12,23^. In the current study, adolescents had higher LPP than adults during emotion regulation in all time windows. Also, for adolescents, the regulation effect was larger when using up-regulation strategy than down-regulation strategy in the late time window. As stated before, late LPP was the indicator of the involvement of cognitive resources and effective cognitive regulation. Thus, the age-related differences between adolescents and adults in late LPP could be a possible evidence of adolescents’ inferior capability in increasing their subjective emotional intensity comparing to adults. Moreover, age-related differences in the relative changes between regulation and no-regulation from the behavioral result suggested that adults were more capable of negative up-regulation and positive down-regulation than adolescents. Taken together, behavioral and neural results of our study both suggest that adults are more effective in up-regulation than adolescents.

However, as mentioned before, in Pitskel’s fMRI study^28^ of up- and down-regulation in children and adolescents (aged from 7 to 17, *M* = 13.03, *SD* = 2.20), there was no age difference of the prefrontal regulatory activation in the emotional up-regulation condition. Given that the age range of our sample includes older participants (*M* = 23.78, *SD* = 2.41) and significantly older than participants in the Pitskel’s study^28^, the contradictory findings from the prior study suggested that adolescents were still developing their ability to use up-regulatory strategies comparing to their older counterparts.

In terms of the down-regulatory strategy, we did not find age differences in the down-regulation effect at either behavioral or neural level. Consistent with our findings, another study on emotion regulation didn’t find any age-related differences in down-regulation between adolescents and adults^28^. However, previous fMRI studies have found age differences in the neural activations in some emotion-related regions. For example, McRae and colleague^38^ found that amygdala was less activated for adolescents compared to adults when asking them to down-regulate their emotion^38^. These results suggest that the ability of down-regulating emotion is still developing for adolescents. However, our result is consistent with another study, which also found no age-related difference in down-regulation between adolescents and adults^28^. These diverge findings may result from the different age ranges or different emotion stimuli. Further studies should examine this issue. Similarly, past findings showed that there was no age-related difference between younger (*M*_*age*_ = 20.7) and older adults (*M*_*age*_ = 68.1)^11^. Taken together, these findings suggest that successful usage of down-regulation might develop before adolescence.

Moreover, different findings on the age differences in up- and down-regulation effect observed in our study suggest that different regulatory strategies might develop in different stages of life. Neuroimaging literature documented that the activated regions during up- and down-regulation are different^39^. Reduced activity in the left dorsolateral prefrontal cortex was observed during down-regulation and enhanced activity in the right parietal cortex was observed during up- regulation^39^. It is likely that the different developmental trajectories of up- and down-regulation effect might originate due to the differences in the developmental maturation of the activated regions for up- and down-regulation. Thus, it is important for future research to examine whether adolescents’ emotion regulation vary as a function of their brain maturation.

As expected, spatial changes over time were found in both age groups, with cortical layer activation from the primary posterior cortical to equally anterior portion. The main effect of Anterior-Posterior was significant in the middle window (700–1000 ms). LPP was larger for posterior recording sites than anterior recording sites. However, the main effect of Anterior-Posterior was not significant in the late window (1000–1500 ms). In the present study, LPP peak was around 600 ms boundary, which was different from the previous study^5^. It implied that the process of emotion regulation might start around this time. It could be a possible reason for posterior activation having not dominated in the early window. Thus, researchers must make careful consideration during the selection of LPP time windows.

Regarding personality differences, we didn’t measure any personality variables in our study. Another important future direction is to identify how personality differences could potentially influence emotion regulation during development. Pictorial stimuli were used in the present study. Although we controlled the size of the images, other visual characteristics (e.g., luminescence and contrast) may influence the ERPs. Thus, it is important for future research to control relevant visual characteristics of the images. In the current study, LPP is generally larger for adolescents compared to adults. Although previous studies demonstrated that the larger ERPs for adolescents could be due to higher activations and immaturity of the social brain^4^. It is likely that the general difference in ERPs between groups may also be because of the thinner skull in adolescents. Because of the thinner skull, the electrodes may capture larger ERPs for adolescents compared to adults. Researchers should take the possible influence of the skull into consideration when trying to understand the development during adolescence.

In summary, this is the first study to examine how emotion regulation modulates late positive potential (LPP) in adolescents. Since adolescents experience increased hormonal changes and developmental challenges, they are more emotionally reactive than children and adults^25^. Consistently, in our study, we observed a higher LPP during emotion regulation in adolescents than in adults. Adolescents showed a higher LPP than adults when using different regulatory strategies in the middle and late time windows. Due to development of the brain socially, neural efficiency improves from adolescence to adulthood^2,38^. Although behavioral and neuroimaging measures suggest that emotion regulation improves with aging^26,40^, few ERP studies to date have examined this notion with the sample of adolescents. In our study, we observed higher amplitude of LPP during emotion regulation processing in adolescents than in adults. In late time window for adolescents, regulation effect was larger when using up-regulation strategy than down-regulation strategy. For adults, however, there was no significant difference between up- and down-regulation. For adolescents, the reactivity effect was larger in negative conditions than in positive conditions in early time window. No significant difference between negative and positive emotional conditions was observed for adults. The excellent temporal sensitivity of LPP allowed us to explore differences between adolescents and adults in different time stages in emotion regulation^39^. These findings provide evidence for the differences in the recruitment of later cognitive mechanisms in emotion regulation between adolescents and adults.

## Supplementary information

Supplementary Materials
