# Supplementary material for: Age-Related Differences in the Late Positive Potential during Emotion Regulation between Adolescents and Adults
Source: Sci Rep. 2019 Apr 5;9:5738. doi: 10.1038/s41598-019-42139-4 (PMC6451025; doi:10.1038/s41598-019-42139-4)
Supplement: Supplementary file 1 — Supplementary Materials [file 41598_2019_42139_MOESM1_ESM.pdf]

Age-Related Differences in the Late Positive Potential during Emotion Regulation between Adolescents and Adults

Xinmei Deng[1, 2], Biao Sang[3, 4 CA], Yixuan Ku[4 CA], Liyang Sai[5]

1. College of Psychology and Sociology, Shenzhen University, Shenzhen, China

2. Shenzhen Key Laboratory of Affective and Social Cognitive Science，Shenzhen, China

3. Shanghai Academy of Educational Science, Shanghai, China

4. School of Psychology and Cognitive Science, East China Normal University, Shanghai, China

5. Hangzhou Normal University, Hangzhou, China

* Corresponding to Biao Sang (bsang@psy.ecnu.edu.cn) or Yixuan Ku(yixuanku@gmail.com).

Dr. Biao Sang or Dr. Yixuan Ku,

Shanghai Academy of Educational Science, Shanghai, China

Supplementary Materials 1:

Table 1. Average Amplitudes of LPP in different experimental conditions and ROIs between adolescents and adults.

Table 2. Results of the repeated measures ANOVA of LPP in different experimental conditions and ROIs between adolescents and adults.

Table 3. Results of the repeated measures ANOVA of reactivity effect in different experimental conditions and ROIs between adolescents and adults.

Table 4. Results of the repeated measures ANOVA of regulation effect in different experimental conditions and ROIs between adolescents and adults.

Figure 7. Voltage scalp topographies of the regulation effects (LPP modulation between regulation condition and no-regulation condition), separately for each time window and age group.

Figure 8. Voltage scalp topographies of the reactivity effects (LPP modulation between positive/negative no-regulation condition and neutral no-regulation condition), separately for each time window and age group.

Supplementary Materials 2:

Table 5. Results of the repeated measures ANOVA of LPP in different experimental conditions and ROIs in adolescents.

Table 6. Results of the repeated measures ANOVA of LPP in different experimental conditions and ROIs in adults.

Table 7. Results of the repeated measures ANOVA of reactivity effect in different experimental conditions and ROIs in adolescents.

Table 8. Results of the repeated measures ANOVA of reactivity effect in different experimental conditions and ROIs in adults.

Table 9. Results of the repeated measures ANOVA of regulation effect in different experimental conditions and ROIs in adolescents.

Table 10. Results of the repeated measures ANOVA of regulation effect in different experimental conditions and ROIs in adults.

Table 1. Average Amplitudes of LPP in different experimental conditions and ROIs between adolescents and adults.

ROIs	Valence	Regulation Strategy	LPP400-700		LP700-1000		LPP1000-1500	
			Adolescents	Adults	Adolescents	Adults	Adolescents	Adults
LA	Positive	Up-re	5.32（2.71）	3.24（1.57）	2.72（1.13）	1.67（0.78）	2.53（0.88）	1.56（0.69）
		Down-re	5.41（2.13）	3.02（1.18）	2.82（1.32）	1.89（0.85）	2.10（0.99）	1.90（0.88）
		No-re	5.48（2.59）	3.14（1.39）	3.11（3.08）	1.81（0.89）	3.44（4.47）	2.05（1.05）
	Negative	Up-re	7.05（2.80）	4.59（2.30）	4.07（2.67）	2.54（1.30）	4.43（3.92）	1.98（0.82）
		Down-re	7.19（2.89）	4.47（2.19）	4.08（2.53）	2.80（2.10）	2.93（2.21）	1.94（1.26）
		No-re	7.06（3.08）	3.94（1.79）	3.83（2.39）	2.12（1.25）	3.12（2.02）	1.70（0.79）
	Neutral	Up-re	4.62（2.56）	2.31（1.43）	2.74（1.60）	1.65（0.59）	2.85（2.25）	1.96（0.99）
		Down-re	4.42（2.46）	2.34（0.94）	2.43（1.26）	1.64（0.85）	1.95（1.35）	1.58（0.68）
		No-re	4.81（3.10）	2.33（0.82）	2.85（2.55）	1.48（0.56）	2.70（1.92）	1.66（0.98）
		Up-re	3.98（2.35）	1.88（1.09）	2.21（1.40）	1.52（0.83）	2.17（1.42）	1.40（0.82）

	Positive	Down-re	3.80（2.07）	1.94（0.95）	2.34（1.22）	1.48（0.81）	2.05（1.21）	1.53（0.93）
		No-re	4.01（1.74）	1.93（1.22）	2.47（1.53）	1.68（1.41）	2.42（2.01）	1.81（1.56）
		Up-re	5.85（2.96）	2.88（1.81）	3.46（1.67）	1.86（1.03）	2.89（1.52）	1.56（1.10）
MA	Negative	Down-re	5.20（2.35）	2.78（1.27）	3.02（1.77）	2.23（1.86）	2.16（1.24）	2.11（1.79）
		No-re	5.27（2.42）	2.42（0.85）	2.95（1.95）	1.79（1.28）	2.77（2.83）	2.05（1.42）
		Up-re	3.68（1.92）	1.72（0.81）	2.14（1.29）	1.61（0.86）	2.46（1.61）	1.79（0.86）
	Neutral	Down-re	3.61（2.17）	1.63（0.65）	2.28（1.43）	1.54（0.82）	2.02（1.71）	1.54（0.98）
		No-re	3.43（1.57）	1.74（0.82）	1.89（0.98）	1.45（0.77）	2.18（1.42）	1.60（0.67）
		Up-re	5.18（2.75）	2.93（1.57）	2.89（1.84）	1.68（1.06）	2.49（1.07）	1.42（0.85）
	Positive	Down-re	4.90（2.33）	2.67（1.50）	2.84（1.86）	1.66（0.65）	2.28（1.24）	1.67（0.67）
		No-re	5.24（2.76）	2.94（1.92）	2.92（2.80）	1.93（1.13）	2.70（2.51）	1.92（1.05）
		Up-re	6.62（2.84）	3.66（2.17）	3.87（2.72）	2.30（1.08）	3.81（3.03）	2.04（0.98）
RA	Negative	Down-re	6.33（2.66）	3.37（1.66）	3.58（2.12）	2.25（1.30）	2.40（1.35）	1.91（0.74）

		No-re	6.40（3.08）	3.24（1.29）	3.75（2.20）	1.99（1.05）	2.64（1.43）	1.98（1.09）
		Up-re	3.95（2.03）	2.38（1.58）	2.56（1.64）	2.04（1.43）	2.66（1.06）	2.24（1.44）
	Neutral	Down-re	3.78（1.52）	2.13（0.96）	2.37（0.98）	1.77（0.64）	2.31（1.39）	1.78（0.59）
		No-re	4.02（1.48）	2.39（1.19）	2.37（1.09）	1.71（0.80）	2.74（1.64）	1.89（1.04）
		Up-re	5.57（2.61）	3.51（1.80）	3.52（2.65）	1.98（1.29）	2.76（1.71）	1.63（1.02）
	Positive	Down-re	4.98（1.86）	3.23（1.46）	3.10（1.79）	1.82（0.98）	2.45（1.07）	1.44（0.73）
		No-re	4.93（1.89）	3.49（2.00）	2.89（1.33）	1.94（1.35）	2.53（1.35）	1.56（1.06）
		Up-re	6.68（2.84）	4.16（2.03）	3.49（1.84）	2.26（1.30）	2.89（1.55）	1.60（0.88）
LP	Negative	Down-re	6.59（2.06）	3.82（1.82）	4.01（1.54）	2.46（1.56）	2.67（1.43）	1.90（0.98）
		No-re	6.23（2.43）	3.97（1.53）	3.44（1.64）	2.29（1.08）	2.49（0.95）	1.75（0.94）
		Up-re	3.69（1.81）	2.47（1.53）	2.39（1.11）	1.73（1.19）	2.46（1.18）	1.79（0.95）
	Neutral	Down-re	3.81（1.29）	2.20（1.31）	2.32（0.76）	1.46（0.66）	2.16（0.70）	1.30（0.52）
		No-re	4.39（1.68）	2.49（1.31）	2.81（1.52）	1.64（0.83）	2.48（1.49）	1.55（0.92）

		Up-re	4.71（1.81）	2.83（0.97）	3.66（1.76）	2.00（0.78）	3.09（1.90）	1.64（0.67）
	Positive	Down-re	4.46（1.45）	2.74（0.88）	3.67（1.24）	2.00（0.93）	3.28（1.66）	1.74（0.82）
		No-re	4.79（1.40）	3.13（0.99）	3.90（1.90）	2.30（1.10）	3.47（1.86）	2.09（1.04）
		Up-re	4.98（1.28）	2.89（1.12）	3.89（1.60）	2.06（1.90）	3.76（1.92）	1.95（0.83）
MP	Negative	Down-re	4.49（1.29）	2.76（0.98）	3.38（1.81）	2.22（1.06）	2.93（1.68）	2.08（1.21）
		No-re	4.67（1.40）	3.04（1.09）	3.23（1.46）	2.35（1.29）	2.93（1.30）	2.16（1.15）
		Up-re	3.27（1.05）	2.36（0.96）	2.57（1.03）	1.83（0.81）	2.81（1.38）	1.89（0.75）
	Neutral	Down-re	3.10（0.97）	1.98（1.07）	1.98（0.85）	1.60（0.61）	2.28（0.90）	1.66（0.63）
		No-re	3.42（1.50）	2.29（0.91）	2.93（1.34）	1.94（0.83）	3.00（1.56）	1.79（0.75）
		Up-re	6.16（2.88）	2.70（1.17）	3.25（2.10）	2.05（1.05）	3.30（2.70）	1.88（1.21）
	Positive	Down-re	6.18（2.49）	2.87（1.42）	3.12（1.40）	1.87（0.90）	3.15（1.54）	1.73（0.89）
		No-re	6.35（2.52）	2.96（1.69）	3.71（2.33）	1.88（0.77）	3.62（3.49）	1.93（0.81）
		Up-re	8.65（3.96）	3.75（2.08）	4.39（2.14）	2.01（0.89）	3.42（1.38）	1.72（0.80）

RP	Negative	Down-re	8.24（3.28）	3.50（1.61）	4.24（1.95）	2.29（1.07）	3.14（1.78）	2.08（0.84）
		No-re	8.00（3.75）	3.55（1.66）	3.97（2.32）	2.14（0.84）	3.21（1.57）	1.80（0.88）
		Up-re	5.07（2.54）	2.19（0.90）	2.77（1.64）	2.01（0.97）	2.60（1.45）	2.19（0.99）
	Neutral	Down-re	4.73（2.34）	2.16（0.63）	2.07（0.76）	1.70（0.69）	2.06（0.88）	1.60（0.56）
		No-re	5.09（1.91）	2.16（0.94）	3.27（1.81）	1.92（0.95）	3.15（1.89）	2.02（1.19）

Table 2. Results of the repeated measures ANOVA of LPP in different experimental conditions and ROIs between adolescents and adults.

Time Window	Factor	*F*	*p*	η_p^2	Pos hoc				
**LPP 400-700**	Age	30.38	.001	.49	Adolescents > Adults ***				
	Laterality	17.80	.001	.36	Left > Medial***<br>Right > Medial***				
	Brain Regions	4.06	.052	.11	Posterior > Anterior				
	Valence	82.53	.001	.72	Neg > Pos ***<br>Neg > Neu***<br>Pos > Neu***				
	Regulation Strategy	1.47	.239	.04					
	Age × Laterality	3.94	.026	.11	**Adolescents**<br>Left > Medial**<br>Right > Medial***	**Adults**<br>Left > Medial*	**Left**<br>Adolescents > Adults***	**Medial**<br>Adolescents > Adults ***	**Right**<br>Adolescents > Adults***
	Age × Brain Regions	.06	.804	.00					
	Age × Valence	7.06	.004	.18	**Adolescents**<br>Pos > Neu***<br>Neg > Pos***<br>Neg > Neu***	**Adults**<br>Pos > Neu**<br>Neg > Pos***<br>Neg > Neu***	**Positive**<br>Adolescents > Adults***	**Negative**<br>Adolescents > Adults***	
	Age × Regulation Strategy	.04	.947	.00					
	Laterality × Brain Regions	2.19	.130	.06					
	Laterality × Valence	13.84	.001	.30			**Left**<br>Pos > Neu***	**Medial**<br>Pos > Neu***	**Right**<br>Pos > Neu***

						Neg > Pos***<br>Neg > Neu***	Neg > Pos***<br>Neg > Neu***	Neg > Pos***<br>Neg > Neu***
Laterality × Regulation Strategy	.20	.908	.01					
Brain Regions × Valence	9.41	.001	.22	**Anterior**<br>Pos > Neu***<br>Neg > Pos***<br>Neg > Neu***	**Posterior**<br>Pos > Neu***<br>Neg > Pos***<br>Neg > Neu***	**Positive**<br>Posterior > Anterior***		
Brain Regions × Regulation Strategy	1.30	.282	.04					
Valence × Regulation Strategy	1.13	.338	.03					
Age × Laterality × Brain Regions	3.09	.063	.09					
Age × Laterality × Valence	4.25	.005	.12	**Positive in Adolescents**<br>Left > Medial**,<br>Right > Medial***<br>**Positive in Adults**<br>Left > Medial**<br>Right > Medial**	**Negative in Adolescents**<br>Left > Medial***,<br>Right > Medial***<br>**Negative in Adults**<br>Left > Medial**<br>Left > Right*	**Neutral in Adolescents**<br>Left > Medial**<br>Right > Medial**<br>**Neutral in Adults**<br>No sig.		

Age × Laterality × Regulation Strategy	.79	.512	.02					
Age × Brain Regions × Valence	1.11	.331	.03					
Age × Brain Regions × Regulation Strategy	1.29	.283	.04					
Age × Valence × Regulation Strategy	.10	.942	.00					
Laterality × Brain Regions × Valence	17.97	.001	.35	**Negative in Anterior**<br>Left > Medial**,<br>Left > Right***,<br>Medial > Right*	**Positive in Posterior**<br>Medial > Left***,<br>Right > Left***<br>**Negative in posterior**<br>Medial > Left*,<br>Right > Left***<br>**Neutral in posterior**<br>Right > Left***	**Positive in Left**<br>Anterior > Posterior***<br>**Negative in left**<br>Anterior > Posterior***<br>**Neutral in left**<br>Anterior > Posterior**	**Positive in Right**<br>Posterior > Anterior*<br>**Negative in right**<br>Posterior > Anterior***<br>**Neutral in right**<br>Posterior > Anterior*	**Left Anterior**<br>Neg > Pos***<br>Neg > Neu***<br>Pos > Neu***<br>**Medial Anterior:**<br>Neg > Pos***<br>Neg > Neu***<br>Pos > Neu***<br>**Right Anterior**<br>Neg > Pos***<br>Neg > Neu***<br>**Left Posterior**<br>Neg > Pos***<br>Neg > Neu***<br>**Medial Posterior**<br>Neg > Pos***

				Neg > Neu*** Pos > Neu*** **Right Posterior** Neg > Pos*** Neg > Neu*** Pos > Neu***
Laterality × Brain Regions × Regulation Strategy	.96	.426	.03	
Brain Regions × Valence × Regulation Strategy	.26	.877	.01	
Age × Laterality × Brain Regions×Valence	1.10	.359	.03	
Age × Laterality × Brain Regions×Regulation Strategy	.31	.850	.01	
Age × Laterality× Valence × Regulation Strategy	.98	.427	.03	
Age × Brain Regions × Valence × Regulation Strategy	2.30	.074	.07	
Laterality × Brain Regions × Valence × Regulation Strategy	.57	.739	.02	
Age × Laterality × Brain Regions × Valence × Regulation Strategy	.33	.905	.01	

**LPP 700-1000**	Age	17.40	.001	.35	Adolescents > Adults ***				
	Laterality	1.64	.207	.05					
	Brain Regions	4.56	.041	.13	Posterior > Anterior *				
	Valence	27.17	.001	.46	Neg > Pos***<br>Neg > Neu***<br>Pos > Neu**				
	Regulation Strategy	.32	.709	.01					
	Age × Laterality	.46	.588	.01					
	Age × Brain Regions	.90	.351	.03					
	Age × Valence	4.57	.022	.13	**Adolescents**<br>Pos > Neu**<br>Neg > Pos**<br>Neg > Neu**	**Adults**<br>Neg > Pos*<br>Neg > Neu*	**Positive**<br>Adolescents > Adults**	**Negative**<br>Adolescents > Adults***	**Neutral**<br>Adolescents > Adults**
	Age × Regulation Strategy	.35	.689	.01					
	Laterality × Brain Regions	2.86	.065	.08					
	Laterality × Valence	2.36	.092	.07					
	Laterality × Regulation Strategy	1.32	.270	.04					
	Brain Regions × Valence	9.69	.001	.23	**Anterior**<br>Neg > Pos***<br>Neg > Neu***	**Posterior**<br>Pos > Neu**<br>Neg > Pos*<br>Neg > Neu***	**Positive**<br>Posterior > Anterior***		
	Brain Regions × Regulation	1.95	.155	.06					

Strategy								
Valence × Regulation Strategy	1.33	.266	.04					
Age × Laterality × Brain Regions	.55	.582	.02					
Age × Laterality × Valence	1.83	.161	.05					
Age × Laterality × Regulation Strategy	.94	.429	.03					
Age × Brain Regions × Valence	2.13	.134	.06					
Age × Brain Regions × Regulation Strategy	.05	.945	.00					
Age × Valence × Regulation Strategy	.42	.764	.01					
Laterality × Brain Regions × Valence	1.43	.239	.04					
Laterality × Brain Regions× Regulation Strategy	.61	.634	.02					
Brain Regions × Valence × Regulation Strategy	2.83	.033	.08	**Up-ER Positive**<br>Posterior > Anterior***<br>**Down-ER Positive**<br>Posterior > Anterior**	**No-ER Neutral**<br>Posterior > Anterior*	**Up-ER in Anterior**<br>Neg > Pos**, Neg > Neu**<br>**Down-ER in Anterior**<br>Neg > Pos**<br>Neg > Neu***	**Up-ER in Posterior**<br>Neg > Neu**<br>**Down-ER in Posterior**<br>Pos > Neu***<br>Neg > Pos**<br>Neg > Neu***	**Neutral in Posterior**<br>No-ER > Down-ER*

					**No-ER Positive** Posterior > Anterior *	**No-ER in Anterior** Neg > Neu**
	Age × Laterality × Brain Regions×Valence	.86	.465	.03		
	Age × Laterality × Brain Regions×Regulation Strategy	1.05	.465	.03		
	Age × Laterality × Valence × Regulation Strategy	.93	.466	.03		
	Age × Brain Regions × Valence × Regulation Strategy	2.06	.099	.06		
	Laterality ×Brain Regions × Valence ×Regulation Strategy	.86	.516	.03		
	Age × Laterality × Brain Regions × Valence × Regulation Strategy	.72	.617	.02		
**LPP 1000-1500**	Age	15.03	.001	.32	Adolescents > Adults ***	
	Laterality	.86	.404	.03		
	Brain Regions	1.67	.206	.05		
	Valence	4.96	.010	.13	Neg > Neu*	
	Regulation Strategy	3.93	.029	.11	Up-ER > Down-ER*	

Age × Laterality	.09	.861	.00
Age × Brain Regions	1.77	.193	.05
Age × Valence	1.91	158	.06
Age × Regulation Strategy	2.34	.111	.07
Laterality × Brain Regions	.76	.431	.02
Laterality × Valence	.59	.614	.02
Laterality×Regulation Strategy	1.00	.392	.03
Brain Regions × Valence	3.27	.058	.09
Brain Regions × Regulation Strategy	.61	.534	.02
Valence×Regulation Strategy	1.37	.260	.04
Age × Laterality × Brain Regions	1.25	.294	.04
Age×Laterality ×Valence	1.53	.213	.05
Age × Laterality × Regulation Strategy	.41	.729	.01
Age × Brain Regions × Valence	1.98	.158	.06
Age × Brain Regions × Regulation Strategy	1.64	.204	.05
Age × Valence × Regulation Strategy	1.11	.348	.03
Laterality × Brain Regions × Valence	1.38	.253	.04
Laterality × Brain Regions × Regulation Strategy	.85	.478	.03

Brain Regions × Valence × Regulation Strategy	2.14	.104	.06
Age × Laterality × Brain Regions×Valence	1.22	.307	.04
Age × Laterality × Brain Regions×Regulation Strategy	1.00	.399	.03
Age × Laterality × Valence × Regulation Strategy	.38	.866	.01
Age × Brain Regions × Valence ×Regulation Strategy	.33	.795	.01
Laterality ×Brain Regions × Valence ×Regulation Strategy	2.13	.067	.06
Age × Laterality × Brain Regions × Valence × Regulation Strategy	.43	.824	.01

Note: $^{*}p< .05$, $^{**}p< .01$, $^{***}p< .001$. Up-ER = up-regulation , Down-ER = down-regulation, No-ER = no-regulation, Pos = Positive, Neg = Negative, Neu = Neutral.

Table 3. Results of the repeated measures ANOVA of reactivity effect in different experimental conditions and ROIs between adolescents and adults.

Time Window	Factor	*F*	*p*	η_p^2	Pos hoc		
**LPP**	Age	1.70	.202	.05			
**400-700**	Laterality	4.65	.017	.13	Right > Medial*		
	Brain Regions	1.02	.321	.03			
	Valence	20.74	.001	.40	Neg > Pos ***		
	Age × Laterality	4.21	.024	.12	**Adolescents**<br>Right > Left**<br>Right > Medial*	**Adults**<br>Left > Medial*	
	Age × Brain Regions	.56	.459	.02			
	Age × Valence	4.26	.047	.18	**Adolescents**<br>Neg > Pos***	**Adults**<br>No sig.	
	Laterality × Valence	8.24	.001	.20		**Negative**<br>Left > Medial**<br>Right > Medial**	
	Laterality×Brain Regions	.67	.511	.02			
	Brain Regions × Valence	5.07	.031	.13	**Anterior**<br>Neg > Pos***	**Posterior**<br>Neg > Pos**	**Positive**<br>Posterior > Anterior*
	Age × Laterality × Brain Regions	.03	.971	.00			
	Age × Laterality × Valence	1.69	.196	.05			

	Age × Brain Regions × Valence	.52	.476	.02				
	Laterality × Brain Regions × Valence	12.50	.001	.28	**Negative in Left** Anterior > Posterior*	**Negative in Right** Posterior > Anterior**	**Left Anterior** Neg > Pos***, **Medial Anterior** Neg > Pos**	**Left Posterior** Neg > Pos** **Medial Posterior** Neg > Pos** **Right Posterior** Neg > Neu**
	Age × Laterality × Brain Regions× Valence	.88	.416	.03				
**LPP 700-1000**	Age	.86	.362	.03				
	Laterality	.09	.908	.00				
	Brain Regions	1.03	.319	.03				
	Valence	2.35	.135	.07				
	Age × Laterality	1.75	.184	.05				
	Age × Brain Regions	.82	.372	.03				
	Age × Valence	.23	.638	.01				
	Laterality × Valence	2.29	.126	.07				
	Laterality × Brain Regions	.30	.737	.01				
	Brain Regions × Valence	3.23	.081	.09				
	Age × Laterality × Brain Regions	.00	.996	.00				
	Age × Laterality× Valence	.80	.419	.02				
	Age×Brain Regions ×Valence	6.56	.015	.17	**Negative in Adolescents**	**Anterior in Adults**		

					Anterior > Posterior*	Neg > Pos*
	Laterality × Brain Regions × Valence	.75	.459	.02		
	Age × Laterality × Brain Regions× Valence	.49	.583	.02		
**LPP 1000-1500**	Age	.053	.820	.00		
	Laterality	1.29	.279	.04		
	Brain Regions	.68	.417	.02		
	Valence	.08	.774	.00		
	Age × Laterality	.13	.828	.00		
	Age×Brain Regions	.01	.911	.00		
	Age × Valence	.12	.734	.00		
	Laterality × Valence	.42	.580	.01		
	Laterality × Brain Regions	.59	.544	.02		
	Brain Regions × Valence	.44	.511	.01		
	Age × Laterality × Brain Regions	1.06	.349	.03		
	Age × Laterality× Valence	.07	.861	.00		
	Age × Brain Regions × Valence	1.03	.319	.03		
	Laterality × Brain Regions × Valence	2.06	.139	.06		
	Age × Laterality × Brain Regions× Valence	.20	.808	.01		

Note: $^{*}p < .05$, $^{**}p < .01$, $^{***}p < .001$, Pos = Positive, Neg = Negative, Neu = Neutral.

Table 4. Results of the repeated measures ANOVA of regulation effect in different experimental conditions and ROIs between adolescents and adults.

Time Window	Factor	F	p	η_p^2	Pos hoc(Mean)	
**LPP400-700**	Age	.64	.837	.00		
	Laterality	1.20	.304	.04		
	Brain Regions	.96	.335	.03		
	Valence	2.51	.123	.07		
	Regulation Effect	2.32	.137	.07		
	Age × Laterality	.01	.989	.00		
	Age × Brain Regions	8.07	.008	.20	**Adolescents**	**Adults**
					No sig.	Anterior > Posterior**
	Age × Valence	.01	.936	.00		
	Age × Regulation Effect	.13	.723	.00		
	Laterality × Brain Regions	.82	.437	.03		
	Laterality × Valence	.43	.638	.01		
	Laterality ×Regulation Effect	.12	.868	.00		
	Brain Regions × Valence	.73	.400	.02		
	Brain Regions × Regulation Effect	.29	.596	.01		
	Valence × Regulation Effect	.16	.696	.01		
	Age ×Laterality× Brain Regions	.31	.713	.01		
	Age × Laterality ×Valence	.70	.492	.02		
	Age × Laterality × Regulation Effect	2.27	.118	.07		
	Age × Brain Regions × Valence	1.16	.289	.04		
	Age × Brain Regions × Regulation Effect	.29	.592	.01		
	Age × Valence × Regulation Effect	.00	.999	.00		
	Laterality × Brain Regions × Valence	.30	.706	.01		

	Laterality × Brain Regions × Regulation Effect	1.56	.222	.05
	Brain Regions × Valence × Regulation Effect	.02	.878	.00
	Age × Laterality × Brain Regions ×Valence	.05	.923	.00
	Age × Laterality × Brain Regions × Regulation Effect	.37	.641	.01
	Age × Laterality × Valence × Regulation Effect	.71	.472	.02
	Age × Brain Regions × Valence × Regulation Effect	.40	.533	.01
	Laterality × Brain Regions × Valence × Regulation Effect	.66	.510	.02
	Age × Laterality × Brain Regions × Valence × Regulation Effect	.19	.819	.01
**LPP700-1000**	Age	.01	.913	.00
	Laterality	1.53	.226	.05
	Brain Regions	.04	.852	.00
	Valence	2.93	.097	.08
	Regulation Effect	.01	.947	.00
	Age × Laterality	.73	.486	.02
	Age × Brain Regions	1.76	.194	.05
	Age × Valence	.11	.738	.00
	Age × Regulation Effect	.79	.379	.02
	Laterality × Brain Regions	.24	.774	.01
	Laterality × Valence	.18	.799	.01
	Laterality ×Regulation Effect	1.30	.279	.04
	Brain Regions × Valence	.84	.366	.03
	Brain Regions × Regulation Effect	.15	.702	.01
	Valence × Regulation Effect	.09	.765	.00

	Age × Laterality × Brain Regions	.70	.496	.02		
	Age × Laterality × Valence	.56	.549	.02		
	Age × Laterality × Regulation Effect	.70	.489	.02		
	Age × Brain Regions × Valence	1.94	.173	.06		
	Age × Brain Regions × Regulation Effect	.03	.858	.00		
	Age × Valence × Regulation Effect	.42	.520	.01		
	Laterality × Brain Regions × Valence	.93	.382	.03		
	Laterality × Brain Regions × Regulation Effect	.20	.798	.01		
	Brain Regions × Valence × Regulation Effect	1.65	.208	.05		
	Age × Laterality × Brain Regions ×Valence	1.29	.279	.04		
	Age × Laterality × Brain Regions × Regulation Effect	.13	.858	.00		
	Age × Laterality × Valence × Regulation Effect	2.18	.129	.06		
	Age × Brain Regions × Valence × Regulation Effect	.26	.614	.01		
	Laterality × Brain Regions × Valence × Regulation Effect	1.12	.327	.03		
	Age × Laterality × Brain Regions ×Valence × Regulation Effect	.45	.620	.01		
**LPP1000-1500**	Age	.01	.936	.00		
	Laterality	.49	.567	.02		
	Brain Regions	.140	.245	.04		
	Valence	1.75	.196	.05		
	Regulation Effect	1.17	.288	.04		
	Age × Laterality	.33	.660	.01		
	Age × Brain Regions	.61	.441	.02		
	Age × Valence	.22	.642	.01		
	Age × Regulation Effect	7.90	.008	.20	**Adolescents**	**Adults**

				Up-ER >Down-ER*	No sig.
Laterality × Brain Regions	.21	.789	.01		
Laterality × Valence	.86	.405	.03		
Laterality × Regulation Effect	1.42	.249	.04		
Brain Regions × Valence	1.14	.294	.03		
Brain Regions × Regulation Effect	.15	.704	.01		
Valence × Regulation Effect	.32	.575	.01		
Age × Laterality × Brain Regions	.57	.553	.02		
Age × Laterality ×Valence	.63	.501	.02		
Age × Laterality × Regulation Effect	.43	.611	.01		
Age × Brain Regions × Valence	.00	.980	.00		
Age × Brain Regions × Regulation Effect	2.74	.108	.08		
Age × Valence × Regulation Effect	1.98	.170	.06		
Laterality × Brain Regions × Valence	2.53	.099	.07		
Laterality × Brain Regions × Regulation Effect	1.16	.317	.04		
Brain Regions × Valence × Regulation Effect	1.27	.268	.04		
Age × Laterality× Brain Regions ×Valence	.76	.448	.02		
Age ×Laterality × Brain Regions × Regulation Effect	.58	.547	.02		
Age × Laterality × Valence × Regulation Effect	.54	.527	.02		
Age × Brain Regions × Valence × Regulation Effect	.02	.885	.001		
Laterality × Brain Regions × Valence × Regulation Effect	3.91	.031	.11	**Up-ER Negative in Left** Anterior > Posterior*	
Age × Laterality × Brain Regions × Valence × Regulation Effect	.27	.733	.01		

Note: *p< .05, **p< .01, ***p< .001. Up-ER = up-regulation, Down-ER = down-regulation, No-ER = no-regulation.

Figure 7. Voltage scalp topographies of the regulation effects (LPP modulation between regulation condition and no-regulation condition), separately for each time window and age group.

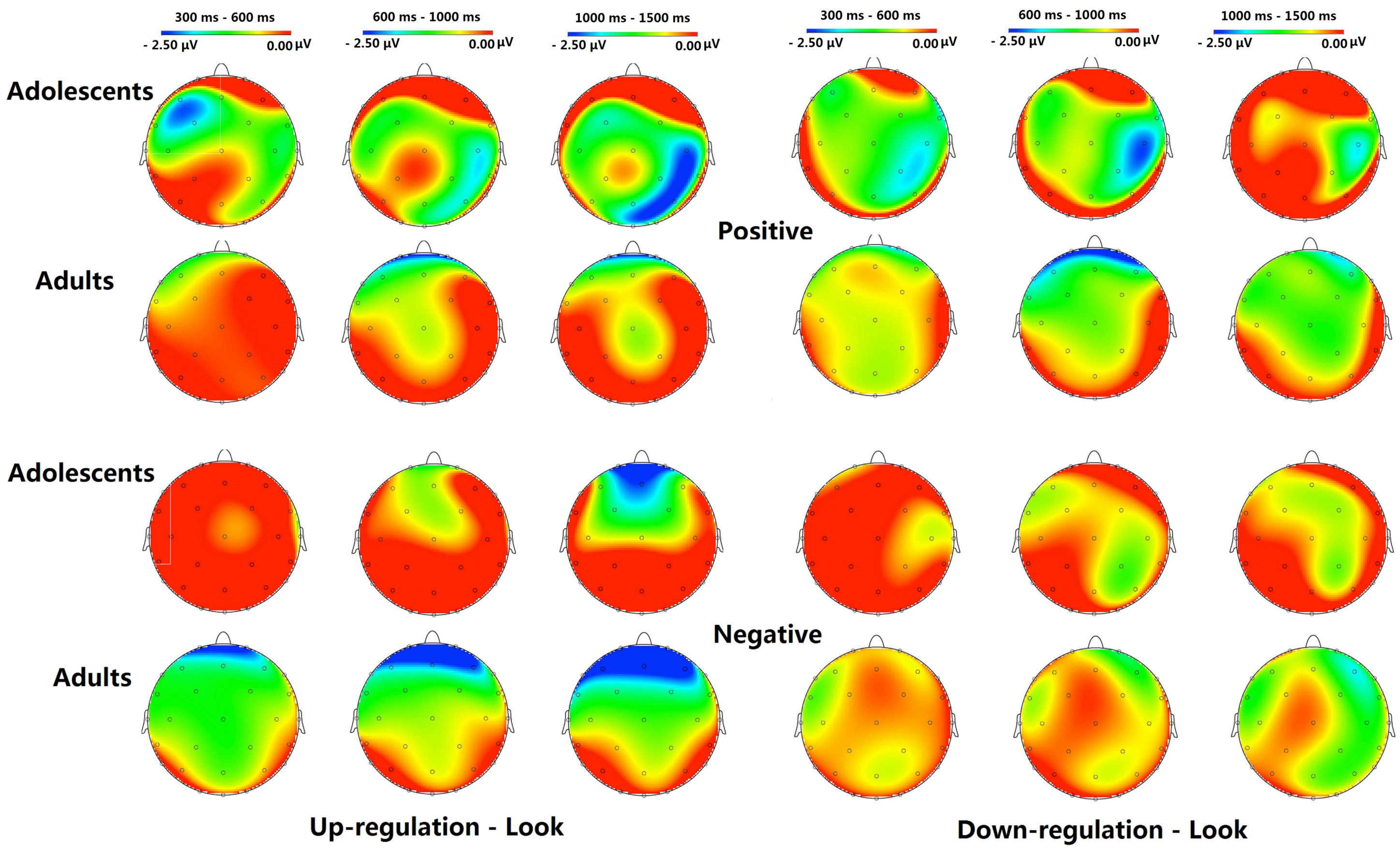

Figure 8. Voltage scalp topographies of the reactivity effects (LPP modulation between positive/negative no-regulation condition and neutral no-regulation condition), separately for each time window and age group.

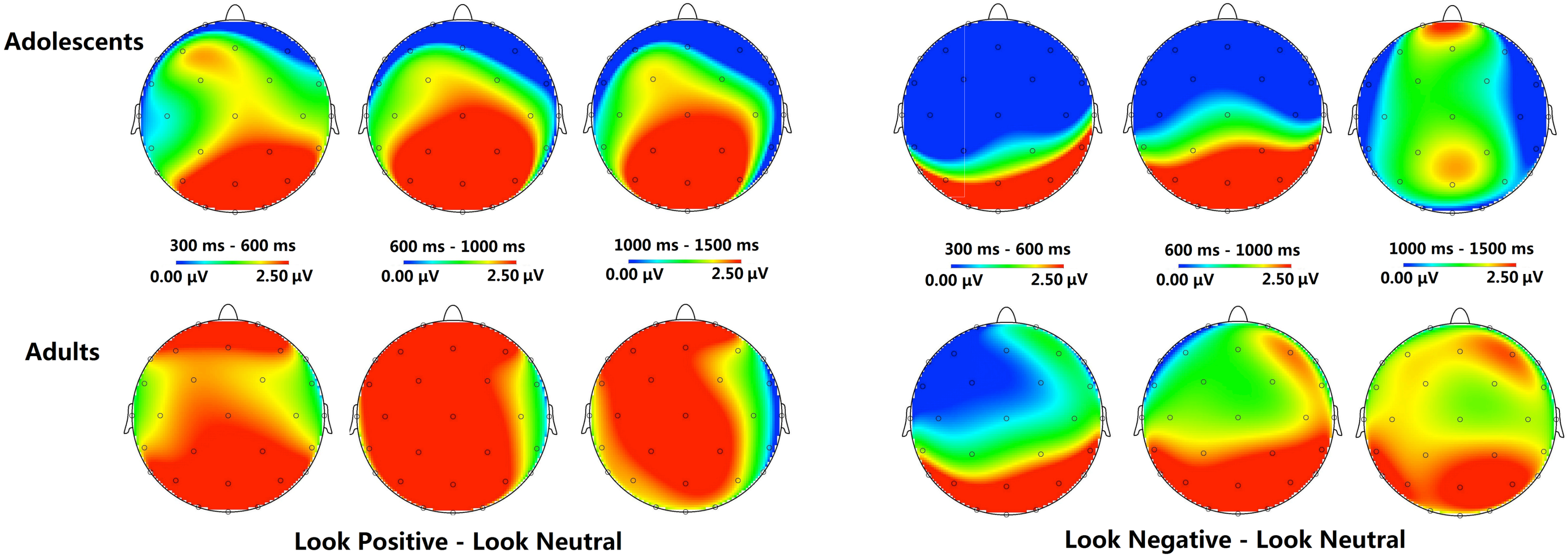

Table 5. Results of the repeated measures ANOVA of LPP in different experimental conditions and ROIs in adolescents.

Time Window	Factor	*F*	*p*	η_p^2	Pos hoc				
**LPP**	Laterality	10.31	.001	.41	Left > Medial*				
**400-700**					Right > Medial**				
	Brain Regions	1.80	.199	.11					
	Valence	43.08	.000	.74	Neg > Pos ***				
					Neg > Neu***				
					Pos > Neu**				
	Regulation Strategy	.65	.515	.04					
	Laterality × Brain Regions	3.25	.068	.18					
	Laterality × Valence	8.31	.000	.36	**Positive**	**Negative**	**Left**	**Medial**	**Right**
					Left > Medial**	Left > Medial**	Pos > Neu**	Pos > Neu**	Pos > Neu**
						Right > Medial**	Neg > Pos***	Neg > Pos**	Neg > Pos***
						**Neu**	Neg > Neu***	Neg > Neu***	Neg > Neu***
						Right > Medial*			
	Laterality×Regulation Strategy	.40	.787	.03					
	Brain Regions × Valence	4.40	.027	.23	**Anterior**	**Posterior**	**Positive**		
					Pos > Neu*	Pos > Neu**	Posterior >		
					Neg > Pos***	Neg > Pos***	Anterior*		
					Neg > Neu***	Neg > Neu***			

Brain Regions × Regulation Strategy	.12	.875	.01					
Valence ×Regulation Strategy	.46	.653	.03					
Laterality × Brain Regions × Valence	3.87	.014	.21	**Negative in Anterior**<br>Left > Medial*<br>**Positive in Posterior**<br>Right > Medial*<br>**Negative in Posterior**<br>Left > Medial**<br>Right > Medial*<br>**Neutral in Posterior**<br>Right > Medial*	**Negative in Right**<br>Posterior > Anterior**<br>**Neutral in Right**<br>Posterior > Anterior**	**Left Anterior**<br>Neg > Pos***<br>Neg > Neu***<br>**Left Posterior**<br>Pos > Neu**<br>Neg > Pos***<br>Neg > Neu***	**Medial Anterior**<br>Neg > Pos**<br>Neg > Neu***<br>**Medial Posterior**<br>Pos > Neu**<br>Neg > Neu***	**Right Anterior**<br>Pos > Neu*<br>Neg > Pos**<br>Neg > Neu***<br>**Righ Posterior**<br>Pos > Neu*<br>Neg > Pos***<br>Neg > Neu***
Laterality × Brain Regions × Regulation Strategy	.30	.835	.02					
Laterality×Valence × Regulation Strategy	1.36	.260	.08					

	Brain Regions × Valence × Regulation Strategy	.94	.429	.06			
	Laterality × Brain Regions × Valence × Regulation Strategy	.50	.758	.03			
**LPP 700-1000**	Laterality	1.48	.246	.09			
	Brain Regions	3.01	.103	.17			
	Valence	14.9	.000	.50	Pos > Neu*<br>Neg > Pos *<br>Neg > Neu**		
	Regulation Strategy	.40	.634	.03			
	Laterality×Brain Regions	2.19	.139	.13			
	Laterality × Valence	1.54	.232	.09			
	Laterality × Regulation Strategy	.90	.443	.06			
	Brain Regions × Valence	6.64	.006	.31	**Anterior**<br>Neg > Pos**<br>Neg > Neu**	**Posterior**<br>Pos > Neu**<br>Neg > Neu**	**Positive**<br>Posterior > Anterior**
	Brain Regions × Regulation Strategy	.47	.594	.03			

	Valence×Regulation Strategy	.68	.570	.04	
	Laterality × Brain Regions × Valence	1.26	.300	.08	
	Laterality × Brain Regions × Regulation Strategy	.69	.574	.04	
	Laterality×Valence × Regulation Strategy	1.06	.385	.07	
	Brain Regions × Valence × Regulation Strategy	2.68	.054	.15	
	Laterality × Brain Regions × Valence ×Regulation Strategy	.79	.547	.05	
**LPP 1000-1500**	Laterality	.36	.614	.02	
	Brain Regions	2.25	.154	.13	
	Valence	3.47	.046	.19	No sig.
	Regulation Strategy	3.73	.042	.20	Up-ER > Down-ER*

Laterality×Brain Regions	3.36	.050	.18	**Posterior** Right > Left*	**Medial** Posterior > Anterior*
Laterality×Valence	.66	.564	.04		
Laterality×Regulation Strategy	.35	.752	.02		
Brain Regions × Valence	2.65	.106	.15		
Brain Regions × Regulation Strategy	1.18	.317	.07		
Valence×Regulation Strategy	.89	.440	.06		
Laterality × Brain Regions × Valence	.25	.247	.09		
Laterality × Brain Regions × Regulation Strategy	.92	.439	.06		
Laterality×Valence × Regulation Strategy	.45	.794	.03		
Brain Regions × Valence ×	1.05	.372	.07		

Regulation Strategy			
Laterality × Brain Regions × Valence ×Regulation Strategy	1.05	.391	.07

Note: $^{*}p < .05$, $^{**}p < .01$, $^{***}p < .001$. Up-ER = up-regulation , Down-ER = down-regulation, No-ER = no-regulation, Pos = Positive, Neg = Negative, Neu = Neutral.

Table 6. Results of the repeated measures ANOVA of LPP in different experimental conditions and ROIs in adults.

Time Window	Factor	*F*	*p*	η_p^2	Pos hoc				
**LPP 400-700**	Laterality	9.96	.000	.37	Left > Medial**				
	Brain Regions	2.37	.142	.12					
	Valence	40.07	.000	.70	Neg > Pos **<br>Neg > Neu***<br>Pos > Neu***				
	Regulation Strategy	.89	.391	.05					
	Laterality × Brain Regions	.84	.42	.05					
	Laterality × Valence	12.47	.000	.42	**Positive**<br>Left > Medial**	**Negative**<br>Left > Medial***<br>Left>Right*<br>Right>Medial*	**Left**<br>Pos > Neu***<br>Neg > Pos***<br>Neg > Neu***	**Medial**<br>Pos > Neu**<br>Neg > Pos**<br>Neg > Neu***	**Right**<br>Pos > Neu**<br>Neg > Pos**<br>Neg > Neu***
	Laterality × Regulation Strategy	.67	.538	.04					
	Brain Regions × Valence	6.78	.005	.29	**Anterior**<br>Pos > Neu**<br>Neg > Pos***<br>Neg > Neu***	**Posterior**<br>Pos > Neu***<br>Neg > Pos**<br>Neg > Neu***	**Positive**<br>Posterior > Anterior**		
	Brain Regions × Regulation Strategy	3.48	.049	.17		**No regulation**<br>Posterior > Anterior*			

Valence × Regulation Strategy	1.01	.40	.06					
Laterality× Brain Regions × Valence	2.95	.05	.15	**Positive in Anterior**<br>Left > Medial***<br>Right > Medial*<br>**Negative in Anterior**<br>Left > Medial**<br>**Neutral in Anterior**<br>Left > Medial*,<br>Right > Medial*	**Positive in Medial**<br>Posterior > Anterior**	**Left Anterior**<br>Pos > Neu***<br>Neg > Posl**<br>Neg>Neu***<br>**Left Posterior**<br>Pos > Neu***<br>Neg > Pos**<br>Neg > Neu***	**Medial Anterior**<br>Neg > Posl**<br>Neg > Neu***<br>**Medial Posterior**<br>Pos > Neu***<br>Neg > Neu**	**Right Anterior**<br>Pos > Neu*<br>Neg > Pos**<br>Neg > Neu***<br>**Righ Posterior**<br>Pos > Neu*<br>Neg > Pos**<br>Neg > Neu***
Laterality×Brain Regions × Regulation Strategy	2.50	.074	.13					
Laterality × Valence × Regulation Strategy	.74	.59	.04					
Brain Regions × Valence ×Regulation Strategy	1.87	.153	.10					
Laterality × Brain Regions × Valence × Regulation Strategy	.21	.952	.01					

**LPP 700-1000**	Laterality	.25	.740	.01		
	Brain Regions	1.32	.267	.07		
	Valence	13.79	.001	.45	Neg > Pos ***<br>Neg > Neu**	
	Regulation Strategy	.03	.969	.002		
	Laterality × Brain Regions	.79	.429	.04		
	Laterality × Valence	3.26	.035	.16	**Left**<br>Neg > Pos**<br>Neg > Neu**	**Right**<br>Neg > Pos*
	Laterality × Regulation Strategy	1.5	.231	.08		
	Brain Regions × Valence	3.63	.042	.18	**Anterior**<br>Neg > Pos**<br>Neg > Neu*	**Posterior**<br>Neg > Pos*<br>Neg > Neu**
	Brain Regions ×Regulation Strategy	2.97	.067	.15		
	Valence × Regulation Strategy	1.35	.27	.07		
	Laterality × Brain Regions × Valence	.20	.878	.01		
	Laterality × Brain Regions × Regulation Strategy	1.30	.282	.07		

	Laterality × Valence × Regulation Strategy	.15	.977	.01
	Brain Regions × Valence × Regulation Strategy	1.78	.153	.10
	Laterality ×Brain Regions × Valence × Regulation Strategy	.51	.754	.03
**LPP 1000-1500**	Laterality	.69	.502	.04
	Brain Regions	.001	.971	.00
	Valence	2.00	.152	.11
	Regulation Strategy	.44	.621	.03
	Laterality × Brain Regions	1.56	.230	.08
	Laterality × Valence	1.96	.138	.10
	Laterality × Regulation Strategy	1.59	.204	.09
	Brain Regions × Valence	.34	.705	.02
	Brain Regions ×Regulation Strategy	.34	.712	.02

Valence×Regulation Strategy	2.66	.061	.14
Laterality × Brain Regions × Valence	.70	.579	.04
Laterality × Brain Regions × Regulation Strategy	.18	.886	.01
Laterality × Valence × Regulation Strategy	.43	.820	.03
Brain Regions × Valence × Regulation Strategy	1.59	.198	.09
Laterality ×Brain Regions ×Valence × Regulation Strategy	1.93	.095	.10

Note: $^{*}p < .05$, $^{**}p < .01$, $^{***}p < .001$. Up-ER = up-regulation , Down-ER = down-regulation, No-ER = no-regulation, Pos = Positive, Neg = Negative, Neu = Neutral.

Table 7. Results of the repeated measures ANOVA of reactivity effect in different experimental conditions and ROIs between adolescents.

Time Window	Factor	*F*	*p*	η_p^2	Pos hoc(Mean)/ Simple effect			
**LPP**	Laterality	2.88	.080	.16				
**400-700**	Brain Regions	.02	.898	.001				
	Valence	14.08	.002	.48	Neg > Pos **			
	Laterality × Valence	5.56	.012	.27	**Left**	**Medial**	**Right**	
					Neg > Pos **	Neg > Pos **	Neg > Pos **	
	Laterality × Brain Regions	.27	.745	.02				
	Brain Regions × Valence	3.37	.086	.18				
	Laterality × Brain Regions × Valence	4.16	.033	.22	**Positive in Posterior**	**Left Anterior**	**Medial Anterior**	**Right Anterior**
						Neg > Pos **	Neg > Pos **	Neg > Pos *
					Medial > Left*	**Left Posterior**		**Right Posterior**
					**Neg in Posterior**	Neg > Pos **		Neg > Pos **
					Right > medial*			
**LPP**	Laterality	.60	.546	.04				
**700-1000**	Brain Regions	.99	.335	.06				
	Valence	1.08	.316	.07				
	Laterality × Valence	1.66	.216	.100				
	Laterality × Brain Regions	.06	.938	.00				

	Brain Regions × Valence	7.00	.018	.32	No sig.
	Laterality × Brain Regions × Valence	.71	.454	.05	
**LPP 1000-1500**	Laterality	.26	.711	.02	
	Brain Regions	.22	.644	.02	
	Valence	.10	.755	.01	
	Laterality × Valence	.07	.834	.01	
	Laterality × Brain Regions	.78	.456	.05	
	Brain Regions × Valence	.81	.383	.05	
	Laterality × Brain Regions × Valence	.82	.436	.05	

Note: $^{*}p < .05$, $^{**}p < .01$, $^{***}p < .001$, Pos = Positive, Neg = Negative, Neu = Neutral.

Table 8. Results of the repeated measures ANOVA of reactivity effect in different experimental conditions and ROIs between adults.

Time Window	Factor	*F*	*p*	η_p^{2}	Pos hoc(Mean)/ Simple effect
**LPP 400-700**	Laterality	11.21	.001	.40	Leftt > Medial** Leftt > Right*
	Brain Regions	6.35	.022	.27	Posterior > Anterior*
	Valence	5.62	.030	.25	Neg > Pos *
	Laterality × Valence	3.24	.056	.16	
	Laterality × Brain Regions	.52	.597	.03	
	Brain Regions × Valence	1.66	.215	.09	
	Laterality × Brain Regions × Valence	1.54	.230	.08	
**LPP 700-1000**	Laterality	1.59	.225	.09	
	Brain Regions	.02	.894	.001	
	Valence	1.88	.188	.10	
	Laterality × Valence	.65	.511	.04	
	Laterality × Brain Regions	.77	.449	.04	
	Brain Regions × Valence	.20	.662	.012	
	Laterality × Brain Regions × Valence	.16	.833	.01	

**LPP 1000-1500**	Laterality	2.13	.155	.11
	Brain Regions	1.09	.311	.06
	Valence	.01	.937	.00
	Laterality × Valence	.85	.425	.05
	Laterality × Brain Regions	.44	.629	.03
	Brain Regions × Valence	.15	.704	.009
	Laterality × Brain Regions × Valence	2.21	.132	.12

Note: $^{*}p < .05$, $^{**}p < .01$, $^{***}p < .001$, Pos = Positive, Neg = Negative, Neu = Neutral.

Table 9. Results of the repeated measures ANOVA of regulation effect in different experimental conditions and ROIs between adolescents.

Time Window	Factor	*F*	*p*	η_p^2	Pos hoc(Mean)
**LPP**	Laterality	.38	.652	.03	
**400-700**	Brain Regions	1.13	.304	.07	
	Valence	.87	.367	.06	
	Regulation Effect	1.31	.270	.08	
	Laterality × Brain Regions	.41	.649	.03	
	Laterality × Valence	.82	.446	.05	
	Laterality × Regulation Effect	.95	.384	.06	
	Brain Regions × Valence	.03	.869	.00	
	Brain Regions × Regulation Effect	.42	.526	.03	
	Valence × Regulation Effect	.05	.833	.003	
	Laterality × Brain Regions × Valence	.18	.751	.01	
	Laterality × Brain Regions × Regulation Effect	.78	.42	.05	
	Laterality × Valence × Regulation Effect	1.31	.282	.08	

	Brain Regions × Valence × Regulation Effect	.08	.781	.01
	Laterality × Brain Regions × Valence × Regulation Effect	.37	.686	.02
**LPP 700-1000**	Laterality	.72	.492	.05
	Brain Regions	.38	.546	.03
	Valence	1.44	.248	.09
	Regulation Effect	.39	.540	.03
	Laterality × Brain Regions	.30	.724	.02
	Laterality × Valence	.43	.598	.03
	Laterality × Regulation Effect	1.05	.347	.07
	Brain Regions × Valence	.07	.790	.01
	Brain Regions × Regulation Effect	.02	.904	.00
	Valence × Regulation Effect	.04	.85	.00
	Laterality × Brain Regions × Valence	1.27	.288	.08
	Laterality × Brain Regions × Regulation Effect	.02	.97	.00

	Laterality × Valence × Regulation Effec	2.13	.147	.12	
	Brain Regions × Valence × Regulation Effect	.95	.346	.06	
	Laterality × Brain Regions × Valence × Regulation Effect	.66	.510	.04	
**LPP 1000-1500**	Laterality	.05	.913	.00	
	Brain Regions	.97	.339	.06	
	Valence	.81	.382	.05	
	Regulation Effect	5.80	.029	.28	Up-Er > Down-Er*
	Laterality × Brain Regions	.37	.669	.02	
	Laterality × Valence	.75	.443	.05	
	Laterality × Regulation Effect	.87	.403	.06	
	Brain Regions × Valence	.33	.576	.021	
	Brain Regions × Regulation Effect	1.22	.286	.08	
	Valence × Regulation Effect	.11	.309	.07	
	Laterality × Brain Regions × Valence	1.45	.252	.09	

Laterality × Brain Regions × Regulation Effect	.92	.398	.06
Laterality × Valence × Regulation Effect	.74	.443	.05
Brain Regions × Valence × Regulation Effect	.27	.612	.02
Laterality × Brain Regions × Valence × Regulation Effect	1.51	.241	.09

Note: $^{*}p< .05$, $^{**}p< .01$, $^{***}p< .001$. Up-ER = up-regulation, Down-ER = down-regulation, No-ER = no-regulation.

Table 10. Results of the repeated measures ANOVA of regulation effect in different experimental conditions and ROIs between adults.

Time Window	Factor	*F*	*p*	η_p^2	Pos hoc(Mean)
**LPP 400-700**	Laterality	1.36	.27	.07	
	Brain Regions	12.78	.002	.43	Anterior > Posterior**
	Valence	1.85	.192	.10	
	Regulation Effect	.95	.34	.05	
	Laterality × Brain Regions	1.36	.271	.07	
	Laterality × Valence	.10	.878	.01	
	Laterality × Regulation Effect	1.89	.169	.10	
	Brain Regions × Valence	1.75	.204	.09	
	Brain Regions × Regulation Effect	.00	.996	.00	
	Valence × Regulation Effect	.17	.683	.01	
	Laterality × Brain Regions × Valence	.13	.875	.01	
	Laterality × Brain Regions × Regulation Effect	1.35	.273	.07	

	Laterality × Valence × Regulation Effect	.93	.392	.05	
	Brain Regions × Valence ×Regulation Effect	.46	.507	.03	
	Laterality × Brain Regions × Valence × Regulation Effect	.49	.538	.028	
**LPP**	Laterality	1.88	.176	.10	
**700-1000**	Brain Regions	2.60	.125	.133	
	Valence	1.49	.239	.08	
	Regulation Effect	.40	.537	.02	
	Laterality × Brain Regions	1.78	.190	.10	
	Laterality × Valence	.143	.855	.01	
	Laterality × Regulation Effect	.69	.504	.04	
	Brain Regions × Valence	4.78	.043	.22	**Negative**<br>Anterior > Posterior*
	Brain Regions × Regulation Effect	.24	.63	.01	
	Valence × Regulation Effect	1.15	.298	.06	
	Laterality × Brain Regions × Valence	.141	.859	.01	

	Laterality × Brain Regions × Regulation Effect	.97	.386	.05
	Laterality × Valence × Regulation Effec	.09	.883	.01
	Brain Regions × Valence × Regulation Effect	.69	.418	.04
	Laterality × Brain Regions × Valence × Regulation Effect	.98	.375	.05
**LPP 1000-1500**	Laterality	1.91	.174	.10
	Brain Regions	.37	.550	.02
	Valence	1.62	.221	.09
	Regulation Effect	2.00	.18	.11
	Laterality × Brain Regions	.24	.727	.01
	Laterality × Valence	.30	.703	.02
	Laterality × Regulation Effect	.70	.500	.04
	Brain Regions × Valence	1.60	.223	.09
	Brain Regions × Regulation Effect	1.83	.194	.10

Valence × Regulation Effect	.89	.358	.05		
Laterality × Brain Regions × Valence	1.98	.168	.10		
Laterality × Brain Regions × Regulation Effect	.16	.852	.01		
Laterality × Valence × Regulation Effect	.52	.560	.03		
Brain Regions × Valence × Regulation Effect	2.19	.157	.11		
Laterality × Brain Regions × Valence × Regulation Effect	3.82	.049	.18	**Pos Up-ER in Left** Posterior > Anterior*	**Up-ER in Left Anterior** Neg > Pos**

Note: $^{*}p< .05$, $^{**}p< .01$, $^{***}p< .001$. Up-ER = up-regulation, Down-ER = down-regulation, No-ER = no-regulation.
